# Reduced insulin signaling in neurons induces sex-specific health benefits

**DOI:** 10.1126/sciadv.ade8137

**Published:** 2023-02-22

**Authors:** Maarouf Baghdadi, Tobias Nespital, Andrea Mesaros, Sandra Buschbaum, Dominic J. Withers, Sebastian Grönke, Linda Partridge

**Affiliations:** ^1^ Max-Planck Institute for Biology of Ageing, Cologne, Germany.; ^2^ Institute of Clinical Sciences, Faculty of Medicine, Imperial College London, London, UK.; ^3^ Medical Research Council London Institute of Medical Sciences, London, UK.; ^4^ Institute of Healthy Ageing and Genetics, Evolution and Environment, University College London, London, UK.

## Abstract

Reduced activity of insulin/insulin-like growth factor signaling (IIS) extends health and life span in mammals. Loss of the *insulin receptor substrate 1 (Irs1)* gene increases survival in mice and causes tissue-specific changes in gene expression. However, the tissues underlying IIS-mediated longevity are currently unknown. Here, we measured survival and health span in mice lacking IRS1 specifically in liver, muscle, fat, and brain. Tissue-specific loss of IRS1 did not increase survival, suggesting that lack of IRS1 in more than one tissue is required for life-span extension. Loss of IRS1 in liver, muscle, and fat did not improve health. In contrast, loss of neuronal IRS1 increased energy expenditure, locomotion, and insulin sensitivity, specifically in old males. Neuronal loss of IRS1 also caused male-specific mitochondrial dysfunction, activation of *Atf4*, and metabolic adaptations consistent with an activated integrated stress response at old age. Thus, we identified a male-specific brain signature of aging in response to reduced IIS associated with improved health at old age.

## Teaser

Insulin signalling reduction mediates a male-specific, neuronal, mitochondrial stress response associated with health benefits.

## INTRODUCTION

Human life span has been increasing in many parts of the world for the past two centuries, but healthy life span has not kept pace (*1*). Aging is characterized by a general decline in physiological function and an increased prevalence of multiple age-related diseases, including cancer, cardiovascular, and neurodegenerative diseases. The prevalence of age-related disease shows a clear sex difference in humans, whereby women, on average, live longer but suffer greater age-associated morbidity (*2*). Aging is a malleable process that can be ameliorated by genetic, dietary, and pharmacological interventions, with the potential to delay or even compress age-associated morbidity (*3*). However, the response to longevity interventions is often sex specific (*2*), emphasizing the need to include both sexes in longevity studies.

Reduced activity of the insulin/insulin-like growth factor 1 (IGF1) signaling (IIS) pathway is associated with increased longevity and improved health at old age in animal models, including worms (*4*), flies (*5*), fish (*6*), and mice (*7*–*10*). Because of its high evolutionary conservation, IIS has also been suggested to be involved in human longevity. Recent genome-wide association studies have implicated variants in IIS pathway loci with longevity (*11*), and studies of rare genetic variants have found enrichment of IIS variants in centenarians, suggesting a relationship between IIS and longevity in humans (*12*). The finding that a longevity-associated allele from humans reduces IIS activity in cell culture (*13*) further supports this link. Therefore, understanding how reduced IIS mediates longevity will help to decipher the underlying biological mechanisms of aging and the development of therapeutics for healthy aging in the future.

The IIS network plays a central role in regulating growth, metabolism, and survival. In mammals, intracellular IIS activity is initiated by two receptor tyrosine kinases, the insulin receptor (IR) and IGF1 receptors, which upon ligand binding phosphorylate insulin receptor substrate (IRS) proteins, which are key downstream mediators of pathway activity. While mice have four IRS proteins (IRS1 to IRS4), IRS3 is not present in humans (*14*). Mice globally lacking *Irs1* activity [*Irs1* knockout (Irs1KO)] are long-lived (*15*). In contrast, *Irs2* KO mice showed reduced survival, suggesting a specific function for IRS1 in longevity (*15*). Irs1KO mice were not only long-lived but also showed resistance to a variety of age-related pathologies including adiposity, ulcerative dermatitis, reduced bone volume, motor dysfunction, and age-related glucose intolerance (*15*). However, global reduction of IIS also has drawbacks such as reduced body size, reduced fertility, and metabolic syndrome (*16*). It is unclear in which tissues IRS1 acts to affect longevity and what are the underlying molecular mechanisms.

Transcriptomic analysis in livers of Irs1KO mice has linked altered IIS to mitochondrial function (*15*), and subsequent molecular studies have found that livers of IIS mutant mice exhibit reduced mitochondrial respiration, adenosine 5′-triphosphate (ATP) generation, and membrane potential (*17*). Mitochondria are cellular organelles with a central role in energy production, cellular stress response, and apoptosis. Mitochondrial function has long been associated with health and longevity, and mitochondrial dysfunction can cause complex multitissue diseases, including metabolic and neurodegenerative disorders (*18*, *19*). Activating transcription factor 4 (ATF4) is a key mediator of mitochondrial stress in response to perturbations in mitochondrial proteostasis (*20*). In the liver, ATF4 is up-regulated in response to life-span–extending interventions such as methionine restriction and rapamycin and acarbose treatment (*21*). In addition, different mitochondrial stressors, affecting mitochondrial translation, oxidative phosphorylation (OXPHOS) stability, mitochondrial membrane potential disruption, and impairment of mitochondrial import, activate ATF4, which then up-regulates the expression of cytoprotective genes that result in a down-regulation of mitochondrial respiration and activation of the integrated stress response (ISR) through cellular metabolic rewiring (*20*). Moreover, in response to stress, ATF4 induces *Atf5* (*22*), which activates a program to rescue mitochondrial activity (*23*). These adaptations lead to increased cellular resistance and protect cells from mitochondrial stress and apoptosis. Overexpression of the *Atf4* target gene *Fgf21* can extend life span in mice, suggesting that the ISR might also ameliorate aging in mammals (*24*).

In this study, we addressed whether the benefits observed in Irs1KO mice were due to protection from age-associated decline or instead reflected an age-independent effect. Moreover, we extended the phenotypic characterization of age-related phenotypes to include male Irs1KO mice, which have previously been shown to be long-lived but have not been extensively studied with respect to aging pathology. We also deleted IRS1 specifically in the liver, muscle, fat, and brain tissue of male and female mice and systematically assessed adult survival as well as health parameters in young and old mice. In contrast to global deletion of IRS1, we did not detect life-span extension in the tissue-specific Irs1KO mice. However, we found that neuron-specific IRS1 deletion was unique in increasing energy expenditure (EE), locomotor activity, and insulin sensitivity, specifically in old male mice. Reduced neuronal IIS induced male-specific and age-dependent mitochondrial dysfunction, leading to up-regulation of the ISR in the brain and systemic effects in peripheral tissues.

## RESULTS

### Irs1KO mice had increased life span and health span

The effects of reduced IIS on metabolism and longevity are often sex specific (*2*). For instance, female Irs1KO mice in a C57BL/6J background showed greater life-span extension than male mice (*7*). Mutant females were also healthier than controls at old age (*15*). The health status of mutant males was not reported. The improved health at old age could indicate protection against the effects of aging or reflect an age-independent effect. To address these questions, we measured health parameters in 5-month-old (young) and 16-month-old (old) male and female Irs1KO mice. We set out to perform these experiments using Irs1KO mice in a C57BL/6N background. However, homozygous mutant animals were not born in the expected Mendelian ratio, and only 4% of homozygous Irs1KO animals were retrieved from matings between heterozygous males and females (10 males and 13 females of 510 pups). In an earlier study (*15*), there was also a deficit of Irs1KO pups from double heterozygous matings in the C57BL/6J background, particularly for males. We therefore generated these animals in a C3B6F1 hybrid background in which homozygous animals were born in the expected 25% ratio. We assessed the health of young and old male and female Irs1KO mice in the C3B6F1 hybrid background.

#### Life span

**Fig. 1.**
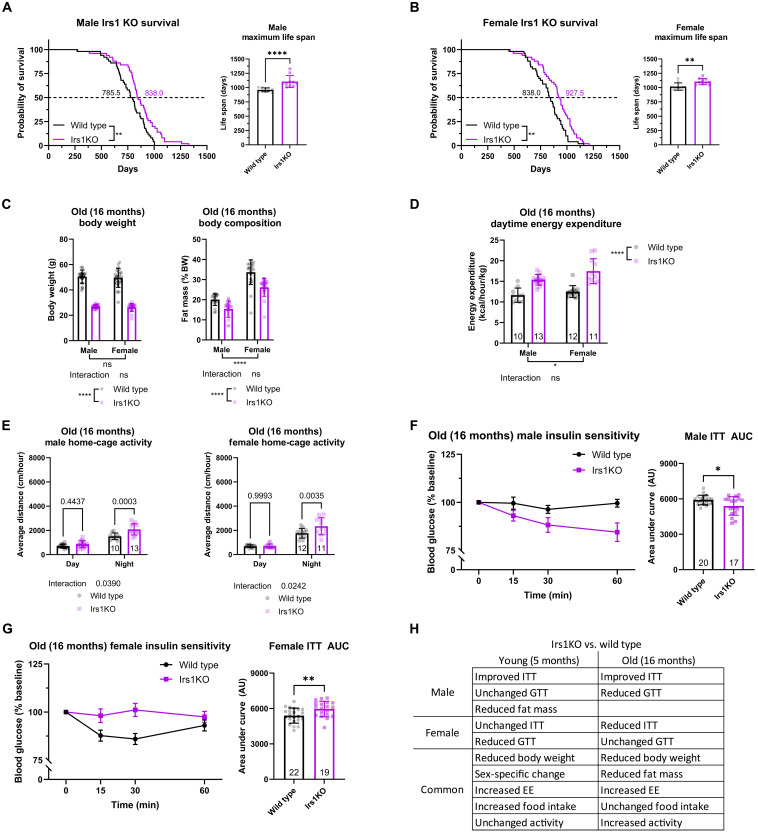
Increased life span and improved health parameters in Irs1KO mice. Kaplan-Meier plots depicting the survival of (**A**) male and (**B**) female wild-type and whole-body Irs1KO mice (*n* = 50 biologically independent animals per sex and genotype). (**C**) Body weight (BW) and composition of Irs1KO mice were measured at old age (16 months) (wild-type males *n* = 20, Irs1KO males *n* = 17, wild-type females *n* = 21, and Irs1KO females *n* = 19). (**D**) Body weight–normalized EE of singly housed old Irs1KO mice during daytime. (**E**) Spontaneous activity of old Irs1KO single-housed mice during daytime (inactive phase) and nighttime (active phase) (wild-type males *n* = 10, Irs1KO males *n* = 13, wild-type females *n* = 12, and Irs1KO females *n* = 11). ITT performed on male (**F**) and female (**G**) Irs1KO mice at old age with respective AUC analysis revealed significantly higher insulin sensitivity in male Irs1KO mice but significantly lower insulin sensitivity in female Irs1KO mice. (**H**) Table summarizing the phenotypes unique to and shared between male and female mutant mice. All error bars correspond to SD except for longitudinal insulin sensitivity where SEM is reported. The number of animals is reported at the bottom of the bars or in the figure legends. Detailed statistical values are found in table S1. GTT, glucose tolerance test; ns, not significant.

To determine whether lack of IRS1 can also extend life span in the C3B6F1 background, we measured the survival of hybrid C3B6F1 Irs1KO mice and their wild-type littermates. Loss of IRS1 led to an extension of median life span by 7 and 11% and of maximum life span, as defined by the 80th percentile age, by 12 and 10% for males (Fig. 1A) and females (Fig. 1B), respectively. The life-span–extending effect of loss of IRS1 is therefore robust to different genetic backgrounds. Moreover, there was no sex bias in life-span extension in the C3B6F1 hybrid background (Cox proportional hazard test, sex × genotype interaction, *P* = 0.4844).

Insulin mutant animals are not only long-lived but also show resistance to age-related diseases (*15*). Given the replication of the Irs1KO life-span extension phenotype in a different mouse genetic background, we assessed whether female hybrid Irs1KO mice also presented with improved age-associated outcomes as reported in the original study (*15*) and extended the characterization to include male Irs1KO mice.

#### Body weight and composition

Irs1KO C3B6F1 mice were dwarves, with a sex-specific reduction in body weight of 46 and 36% in young male and female mice, respectively [two-way analysis of variance (ANOVA), sex × genotype interaction *F*_1,76_ = 22.74, *P* < 0.0001; fig. S1A]. The greater effect of the mutation on male body weight may have been in part attributable to differences in fat content, as young male Irs1KO mice showed a sex-specific reduction in fat mass, with no significant change in females (two-way ANOVA, sex × genotype interaction *F*_1,76_ = 8.76, *P* = 0.0041; fig. S1A). Body weight and fat content were reduced in mutant mice of both sexes at old age, with no significant sex and genotype interaction (two-way ANOVA, body weight sex × genotype interaction term *F*_1,74_ = 0.0144, *P* = 0.9049; fat content sex × genotype interaction term *F*_1,74_ = 1.844, *P* = 0.1787; Fig. 1C). The decreased adiposity of old Irs1KO mice was not due to reduced food intake, because there was no difference in food consumption relative to body weight between old mutant and wild-type animals of either sex (fig. S2A). In contrast, young Irs1KO mice of both sexes ate more food relative to their body weight (fig. S1B) while not showing increased fat mass, suggesting changes in EE.

#### Energy expenditure

Indirect calorimetry showed increased daytime EE in young Irs1KO mice of both sexes (fig. S1C), consistent with the hypothesis that body size and body weight–adjusted EE are inversely correlated (*25*). We also found increased EE in old Irs1KO mice of both sexes during daytime (Fig. 1D) and nighttime (fig. S2B). The decrease in adiposity was therefore probably not mediated by increased EE, as young female Irs1KO mice showed increased EE but no significant difference in adiposity (fig. S1A).

We next measured spontaneous home-cage locomotor activity to investigate whether an increase in movement could account for the decreased adiposity observed in Irs1KO mice. Young male and female Irs1KO mice showed no significant difference in activity levels (fig. S1D). However, there was a notable trend in nighttime male Irs1KO activity (fig. S1D) that may have contributed to the sex-specific reduction in adiposity observed in young male Irs1KO mice (fig. S1A). Analysis of activity levels in old male and female Irs1KO mice revealed a significant increase in nighttime, but not daytime, activity (Fig. 1E). Thus, increased locomotor activity might contribute to the reduced adiposity of old male and female Irs1KO mutant mice, but it cannot explain the reduced fat mass of young male Irs1KO mice.

Consistent with previous findings in female C57BL/6J Irs1KO mice, female hybrid Irs1KO mice developed an age-dependent reduction in adiposity (*15*). However, adiposity presented in a sex-specific manner, where male hybrid Irs1KO mice had significantly decreased fat content independent of age. Moreover, we detected a significant increase in locomotor activity in old male and female Irs1KO mice. One potential explanation could be an amelioration of the age-dependent decrease in locomotor activity observed in wild-type mice (*26*). This is consistent with the original report in C57BL/6J female Irs1KO mice of a delay in age-dependent loss of locomotor coordination on the rotarod (*15*).

#### Peripheral metabolism

IIS is a major regulator of glucose metabolism (*27*), and insulin resistance is a causal factor for several age-related pathologies including obesity, type 2 diabetes, and metabolic syndrome (*28*). Therefore, we assessed whether Irs1KO mutant mice showed age-related changes in glucose metabolism by performing insulin tolerance (ITT) and glucose tolerance tests of young and old Irs1KO mice of both sexes as a readout for insulin sensitivity and pancreatic β cell function, respectively. Area under the curve (AUC) analysis of ITT revealed that male Irs1KO mice showed significantly increased insulin sensitivity compared to controls at both young (fig. S1E) and old age (Fig. 1F). In contrast, loss of *Irs1* had no effect on insulin sensitivity in young females (fig. S1F) but caused insulin insensitivity in old females (Fig. 1G), consistent with the results of female Irs1KO C57BL/6J mice (*15*). There was no difference in glucose clearance in young Irs1KO males (fig. S1G), but old Irs1KO males showed a significantly reduced response to the glucose challenge (fig. S2C). Conversely, young Irs1KO females presented with glucose intolerance (fig. S1H), while there was no significant difference in glucose tolerance in old Irs1KO females (fig. S2D). These findings are consistent with previous reports, which also detected no change in glucose tolerance in 16-month-old female Irs1KO C57BL/6J mice (*15*). Glucose tolerance was improved in 23-month-old female Irs1KO C57BL/6J mice (*15*).

In summary, C3B6F1 hybrid Irs1KO mice showed no sex bias in life span or metabolic health parameters such as reduced adiposity, increased EE, and locomotor activity at old age. However, C3B6F1 Irs1KO males showed a sex-specific benefit in insulin sensitivity.

### Tissue-specific Irs1KO mice generation and validation were successful in the liver, muscle, fat, and neurons but not gut tissue

We next investigated the role of the five major insulin-responsive metabolic organs—the liver, muscle, fat, gut, and nervous system—in mediating the effect of IRS1 deficiency on murine longevity and health. Irs1KO mice in the C3B6F1 hybrid background showed similar phenotypes as previously reported for C57BL/6J Irs1KO mice (*15*). Therefore, we conducted the tissue-specific experiments in the C57BL/6N background, as the LoxP floxed *Irs1* allele (*29*) and the tissue-specific Cre drivers were only available in this background. In contrast to the global deletion of IRS1 in the C57BL/6N background, mice with tissue-specific deletion of IRS1 were born in the expected Mendelian ratio, suggesting that development of the animals was not detrimentally affected.

Liver-specific Irs1KO mice were generated using Alfp-CreT (lKO) (*30*), muscle-specific (targeting skeletal and cardiac muscle tissue) using Ckmm-CreT (mKO) (*31*), fat-specific [targeting white adipose tissue (WAT) and brown adipose tissue (BAT)] using Adipoq-CreT (fKO) (*32*), gut-specific (targeting small and large intestine) using Villin1-CreT (gKO) (*33*), and neuron-specific using Syn1-CreT (nKO) (*34*) mice. Male C57BL/6N mice carrying the corresponding *CreT* transgenic constructs were mated to LoxP floxed *Irs1* (*29*) mutant C57BL/6N females to generate tissue-specific Irs1KO mice (*CreT/+::Irs1fl/fl*) and their corresponding LoxP floxed *Irs1* littermate controls (*Cre+/+::Irs1fl/fl*). We first validated the efficiency of IRS1 depletion by measuring *Irs1* transcript levels by quantitative real-time polymerase chain reaction (qRT-PCR) in the corresponding target tissue (fig. S3, A to F). This was done using old male and female mice to verify that the depletion of IRS1 is stable throughout life. Primers and qRT-PCR conditions were validated with cortical brain samples from global Irs1KO mice (fig. S3A). *Irs1* transcripts were strongly depleted in the liver tissue of lKO (fig. S3B), hindlimb muscle tissue of mKO (fig. S3C), and BAT (subscapular) of fKO (fig. S3D) and partly depleted in the cortical brain tissue of nKO mice (fig. S3E). The partial reduction in the cortex is probably explained by the residual expression of *Irs1* in glial cells, which are not targeted by the Syn1-CreT (*34*). In contrast, we did not detect depletion of *Irs1* transcripts in the small intestine (ileum) of gKO mice (fig. S3F), suggesting that *Irs1* mutant cells were outcompeted by wild-type cells in the gut epithelium during aging. Therefore, gKO mice were excluded from further analysis.

We then tested the specificity of the tissue-specific KO lines by measuring *Irs1* expression levels in untargeted tissues, namely, the brain cortex for lKO, mKO, and fKO (fig. S3G) and liver tissue for nKO (fig. S3H), respectively. There was no significant difference in the expression level of Irs1 in the nontargeted tissues, demonstrating the specificity of the generated tissue-specific KO lines. As IRS1 and IRS2 have been suggested to have redundant functions, we used qRT-PCR to measure *Irs2* levels in the IRS1 tissue-specific KO lines. However, except for a slight up-regulation of *Irs2* transcript levels in the liver of male IKO mice (fig. S3B), there was no compensatory up-regulation of *Irs2* gene expression in any of the tested KO lines (fig. S3, A to E). In summary, the four *Irs1* tissue-specific KO lines had efficient depletion of *Irs1* expression, without unspecific effects in other tissues or compensatory regulation of *Irs2* expression. Thus, these lines were suitable for addressing how tissue-specific depletion of IRS1 affects health and longevity.

### Tissue-specific KO of Irs1 in the liver, muscle, fat, or neurons does not extend life span in mice

#### Life span

**Fig. 2.**
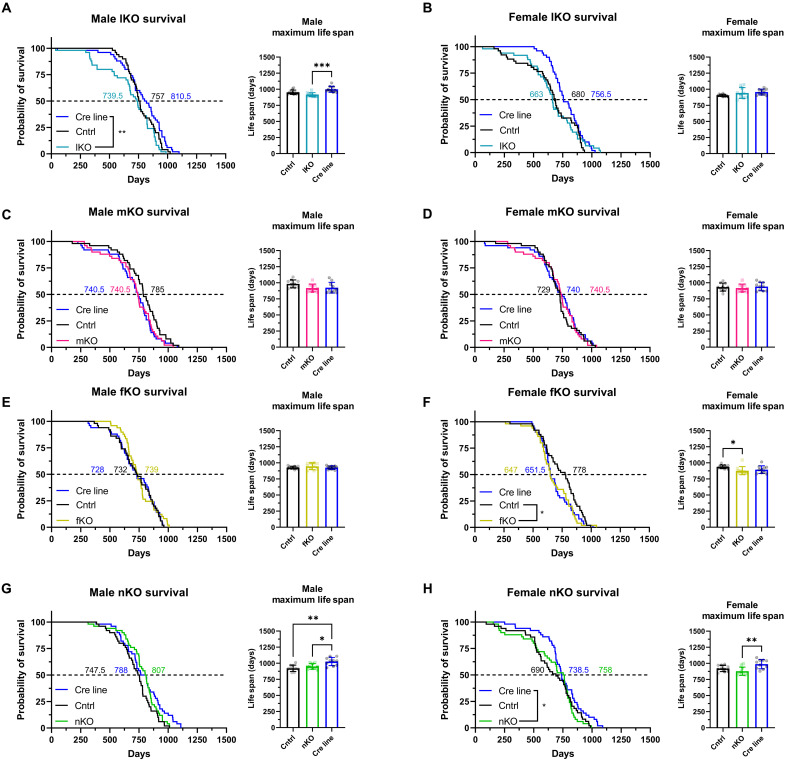
Tissue-specific deletion of IRS1 is not sufficient for life-span extension. Kaplan-Meier plots depicting the survival of male and female mice. Cox proportional hazard tests were used to compare survival (median life span labeled on 50% survival probability line in days) and Kruskal-Wallis tests were used to compare maximum the life span (inset shows the top 20% longest-lived mice) of all mutant mice. (**A**) Male and (**B**) female control (Cntrl), liver-specific *Irs1* KO (lKO), and *AlfpCre* (Cre line) mice (*n* = 50 biologically independent animals for all groups). (**C**) Male and (**D**) female control (Cntrl), muscle-specific *Irs1* KO (mKO), and *CkmmCre* (Cre line) mice (*n* = 50 biologically independent animals for all groups). (**E**) Male and (**F**) female control (Cntrl), fat-specific *Irs1* KO (fKO), and *AdipoqCre* (Cre line) mice (*n* = 50 biologically independent animals for all groups and *n* = 49 for male fKO mice). (**G**) Male and (**H**) female control (Cntrl), neuron-specific *Irs1* KO (nKO), and *Syn1Cre* (Cre line) mice (*n* = 50 biologically independent animals for all groups and *n* = 49 for female control). Detailed statistical values are found in table S1. Raw life-span data are found in table S4.

As global loss of *Irs1* results in life-span extension, we compared the survival of tissue-specific *Irs1* mutant mice to their respective *Cre* and *Irs1fl/fl* control lines to address which tissue might underlie the longevity effect. However, there was no life-span extension detected between mutant mice and floxed littermates by Cox proportional hazard tests in male or female lKO, mKO, fKO, and nKO (Fig. 2) mice. We observed a significant reduction in survival in fKO females compared to their control littermates. However, this reduction in life span may be due to the presence of Adipoq-Cre, as there was no significant difference between the Cre line and fKO females. In summary, we did not detect life-span extension in any of the tested *Irs1* tissue-specific KO lines, suggesting that reduction of IIS in more than one tissue or in a tissue we have not targeted is required for life-span extension in mice.

### IRS1 deletion in the liver, muscle, or fat does not improve health at old age

#### Body weight and composition

To address whether tissue-specific deletion of IRS1 would lead to health benefits, we conducted phenotyping of males and females of the *Irs1* tissue-specific KO lines. In contrast to whole-body Irs1KO animals, which are dwarfs, there was no difference in body weight in young lKO, mKO, or fKO animals (fig. S4, A. E, and I), indicating that the deletion of IRS1 in these tissues did not interfere with overall growth.

The liver stores glucose after a meal as glycogen or converts excess glucose to fatty acids. It also oxidizes fatty acids to provide energy for gluconeogenesis during fasting. Moreover, sex affects liver physiology, with substantial consequences for systemic metabolism (*35*). IRS1 plays a role in muscle growth and in insulin-stimulated glucose transport into muscle in male mice (*36*, *37*), but sex-specific effects of IRS1 in muscle tissue have not been fully characterized. Therefore, we assessed whether muscle contributed to the sex-specific health benefits observed in Irs1KO mice. The contribution of adipose tissue to the physiological insulin response is not certain. However, fat-specific IR KO mice are protected against age-associated glucose intolerance and insulin insensitivity (*38*) and show enhanced life span (*39*). However, the role of a relatively modest IIS reduction in IRS1 deletion and the role of sex have not been assessed.

As we observed sex differences in the metabolic phenotypes of Irs1KO mice, we measured metabolic phenotypes in male and female lKO mice. lKO mice showed a sex-specific reduction in body weight only in old males (two-way ANOVA, sex × genotype interaction *F*_1,45_ = 11.38, *P* = 0.0015; fig. S4C), which was probably due to a sex-specific decrease in fat mass (two-way ANOVA, sex × genotype interaction *F*_1,45_ = 5.132, *P* = 0.0284; fig. S4D). Male and female mKO mice showed no change in body weight (fig. S4, E and G) but a significant increase in fat mass relative to body weight at both young and old age in both sexes (fig. S4, F and H). These results are consistent with the findings using muscle-specific IR KO animals, which also showed increased fat mass and reduced muscle mass (*31*). Although male and female fKO mice showed no change in body weight (fig. S4, I and K), we measured a significant reduction in fat mass at young and old age in both male and female fKO mice (fig. S4, J and L), suggesting that, similar to fat-specific IR KO mice (*38*, *40*), fKO mice have a reduced WAT mass. Changes in body weight and composition were not a result of altered food consumption as no significant differences were observed in young and old lKO, mKO, or fKO animals (fig. S4, M to R). There has been a controversy whether IIS in fat can modulate feeding, as one report found a significant increase in fat-specific IR KO mice (*40*), while another study found no difference (*38*). We did not detect any significant changes in food intake of young or old fKO mice of either sex (fig. S4, Q and R), which might be due to the less severe down-regulation of IIS upon loss of IRS1 compared to the IR.

#### Energy expenditure

There was no significant difference in EE in young or old male or female lKO mice (fig. S5, A to D). IRS1 deficiency in muscle and fat tissue did not lead to a significant difference in EE in young or old male and female mKO and fKO mice (fig. S5, E to L). Spontaneous locomotor activity did not reveal any significant difference in young or old male and female lKO mice (fig. S6, A and B). Young male mKO mice showed increased spontaneous locomotor activity specifically during nighttime (fig. S6C), while no change was observed in mKO females or old mKO males (fig. S6D). Loss of IRS1 in the fat did not affect the spontaneous activity of young or old male or female mice (fig. S6, E and F).

#### Peripheral metabolism

Insulin sensitivity of young or old male and female lKO mice was not changed compared to control animals (fig. S7, A to D). Insulin sensitivity of mKO mice was also not significantly changed (fig. S7, E to H), consistent with data from muscle-specific IR and IGF1R KO mice, which had reduced activated IRS1 levels but no change in insulin sensitivity (*31*, *41*). Young male fKO mice had a significantly reduced insulin sensitivity (fig. S7I), while young females showed no significant difference (fig. S7J). Insulin sensitivity of old male fKO mice was not significantly changed (fig. S7K); however, females had significantly reduced insulin sensitivity (fig. S7L).

Consistent with previous glucose tolerance test studies (*42*), male lKO mice showed reduced glucose tolerance at young (fig. S8A) but not old age (fig. S8C). However, a hyperinsulinemic-euglycemic clamp study in another report found no evidence for any difference in glucose tolerance in young male lKO mice (*43*). In contrast to the males in our study, female lKO mice showed no difference in glucose tolerance at young or old age (fig. S8, B and D). Young male and female mKO animals showed a significant reduction in glucose tolerance (fig. S8, E and F), which was unexpected, given that IR or IRS1 + IRS2 KO mice did not show this effect (*31*, *37*). However, the reduction in glucose tolerance was also observed in old male (fig. S8G) but not in old female mKO mice (fig. S8H). Glucose tolerance was significantly reduced in young female fKO mice (fig. S8J) but was unaffected in young males (fig. S8E). Old female fKO mice were more sensitive to glucose than controls (fig. S8L), but no difference was detected in old males (fig. S8K).

In summary, loss of IRS1 in the liver affected body composition and glucose tolerance in males. Loss of IRS1 in muscle reduced lean mass and glucose tolerance of both males and females at young age, while locomotor activity was increased only in young males. Fat tissue–specific loss of IRS1 function mostly affected peripheral metabolism in females. Thus, loss of IRS1 affects peripheral metabolism in a tissue- and sex-specific manner.

### Neuron-specific deletion of IRS1 causes male-specific improvement in metabolic health

**Fig. 3.**
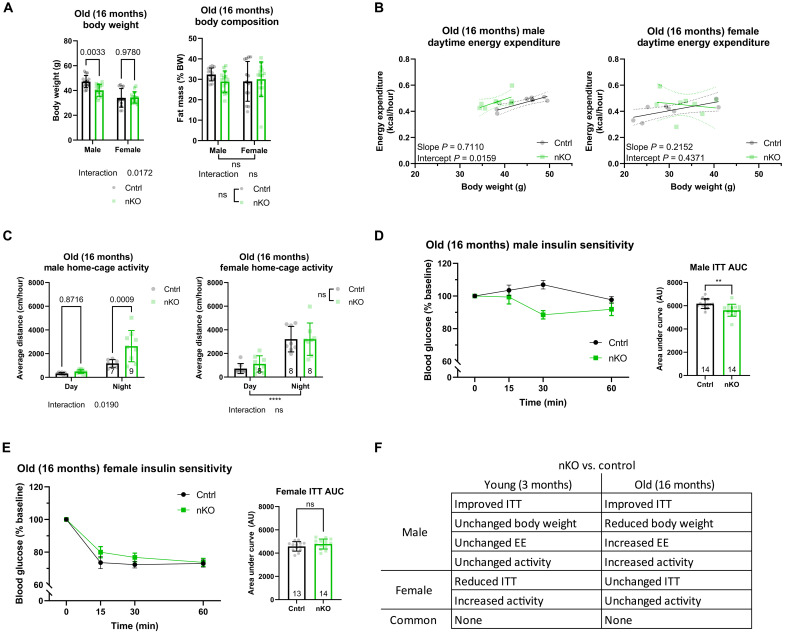
nKO mice show male-specific improvement in metabolic health. (**A**) Body weight and composition of nKO mice were assessed at old age (16 months) (*n* = 14 biologically independent animals for all groups). (**B**) Daytime EE of male (control *n* = 7 and nKO *n* = 8) and female (*n* = 8 female control and nKO) mice was analyzed by linear regression of EE by body weight (ANCOVA). (**C**) Plotted spontaneous home-cage activity of old male (control *n* = 7 and nKO *n* = 9) and female (control *n* = 7 and nKO *n* = 9) single-housed nKO mice showed a nighttime-specific increase in activity of male nKO mice. (**D**) Analysis of ITT curves and AUC values of old male nKO showed a significant improvement in insulin sensitivity in nKO mice compared to controls. (**E**) Analysis of ITT curves and AUC values of old female nKO did not reveal any significant difference compared to controls. (**F**) Table summarizing the phenotypes unique to and shared between male and female mutant mice, highlighting the enrichment of male-specific phenotypes in nKO mice. All error bars correspond to SD except for longitudinal insulin sensitivity where SEM is reported. For ANCOVA analysis, the 95% confidence interval is plotted. The number of animals is reported at the bottom of the bars or in the figure legends. Detailed statistical values are found in table S1.

The brain is a central mediator of metabolic function (*44*), and reduced IIS in the brain has been associated with changes in body weight, fat mass, glucose metabolism, and feeding behavior (*45*). However, peripheral, off-target recombination has been reported in the kidney, pancreas, and muscle of the Cre-driver lines used in these studies (*46*). The Syn1Cre line used here is specific to neurons (*34*) and allowed us to specifically study the effects of reduced IIS in the brain on peripheral metabolism. Therefore, male and female nKO mice at young and old age were phenotyped. There was a slight sex-specific reduction in body weight in old male nKO mice (two-way ANOVA, sex × genotype interaction *F*_1,51_ = 6.069, *P* < 0.0172; Fig. 3A) with no significant change in body composition of either young (fig. S9A) or old nKO (Fig. 3A) mice of either sex. Brain insulin signaling has been implicated in feeding and satiety (*47*). Thus, we measured food consumption in nKO mice but did not detect any differences in young (fig. S9B) or old (fig. S10A) mice of either sex. Previous studies have uncovered a direct role of the brain in mediating EE through peripheral tissues such as BAT (*48*, *49*). In young animals, there was no effect on EE in either male or female nKO mice (fig. S9C). However, EE was specifically increased in old male but not female nKO mice during daytime (Fig. 3B) and nighttime (fig. S10B), indicating that nKO mice show an age-dependent and sex-specific increase in EE. Given the role of neuronal IIS in locomotor activity (*50*), we next measured spontaneous home-cage activity. Locomotor activity was not changed in young nKO males (fig. S9D) but was increased in young females during both daytime and nighttime. In contrast, old nKO males showed a significant increase in activity only during nighttime, while there was no change in old nKO females (Fig. 3C). The male-specific increase in activity was not observed during daytime, suggesting that the effect seen at old age was not due to general hyperactivity or involuntary movements. As neuronal IIS function has been implicated in mediating insulin sensitivity through circuitry with peripheral metabolic organs (*50*), we assessed insulin sensitivity in young and old nKO mice of both sexes. Consistent with male Irs1KO mice, insulin sensitivity was increased in young (fig. S9E) and old (Fig. 3D) male nKO mice. In contrast, young female nKO mice showed a significant reduction in glucose clearance (fig. S9F), which was not present at old age (Fig. 3E). Syn1Cre control mice did not show any significant changes in the corresponding phenotypes, suggesting that the observed differences in nKO mice are not caused by neuronal *Cre* expression (fig. S11, A to J). Thus, IRS1 deletion in neurons was sufficient to induce male-specific benefits in metabolic outcomes that in part recapitulated the phenotypes of the global Irs1KO. Moreover, nKO mice did not show the negative consequences of globally reduced IIS in the C57BL/6 background such as reduced body size, viability, and glucose tolerance (fig. S10, C and D).

In summary, while loss of IRS1 in the liver, muscle, or fat did not improve health at old age, deletion of IRS1 in neurons was sufficient to cause similar health benefits in old males as observed in Irs1KO mice. Thus, reduced neuronal IIS may contribute to the improved insulin sensitivity, increased EE, and locomotor activity of male whole-body Irs1KO mice.

### Loss of IRS1 function caused mitochondrial dysfunction in the brain of old males

**Fig. 4.**
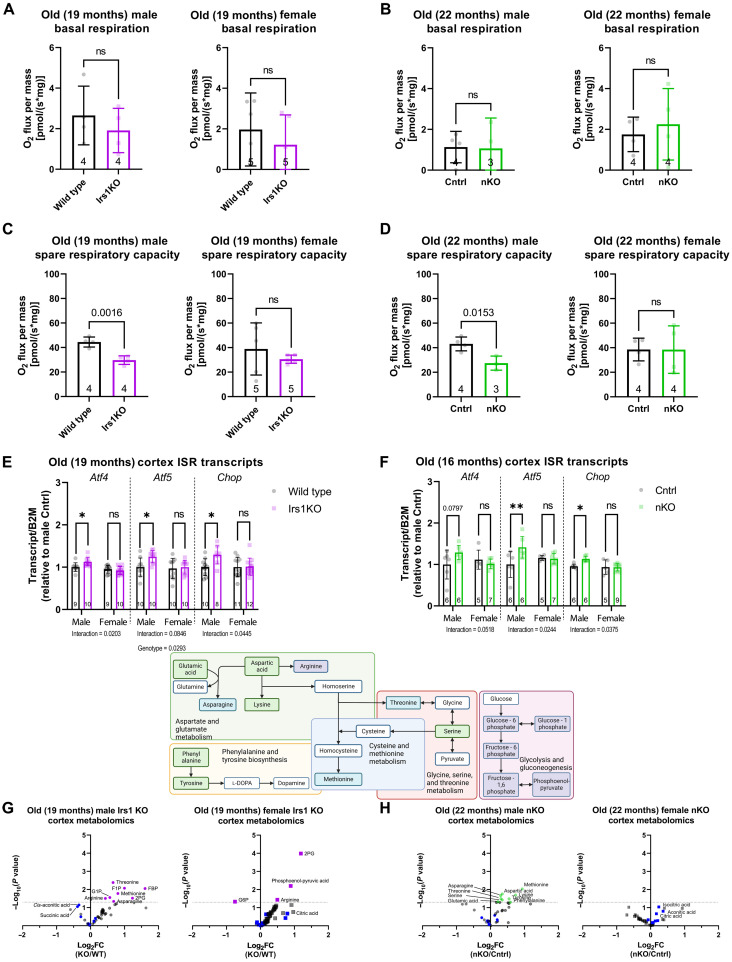
Investigation of mitochondrial function implicated the ISR. (**A**) Measurement of basal oxygen consumption in brain tissue from old (19-month-old) Irs1KO mice detected no differences. (**B**) Measurement of basal oxygen consumption of brain tissue from old (22-month-old) nKO mice did not detect any differences. (**C**) Spare mitochondrial capacity in the brain tissue of old Irs1KO mice was measured, revealing a significant reduction in male but not female Irs1KO brains. (**D**) Spare mitochondrial capacity in the brain tissue of old nKO mice also revealed a significant reduction in male but not female nKO brains. (**E**) qRT-PCR performed to measure ISR markers on the brain tissue of old Irs1KO mice and their wild-type (WT) littermates found a significant sex-specific up-regulation of ISR in male Irs1KO mice. (**F**) Transcripts of ISR markers that were measured in the brain tissue of old (16-month-old) nKO mice and their control littermates found a significant sex-specific up-regulation of ISR in male nKO mice. (**G**) Semitargeted metabolomics revealed up-regulation of some metabolites in old brain tissue of Irs1KO mice compared to littermate wild types (male wild type *n* = 5 and Irs1KO *n* = 6, and female wild type and Irs1KO *n* = 6). Steady-state TCA cycle intermediates are labeled in blue, and the most affected are labeled. (**H**) Metabolomic analysis in old nKO mice also revealed up-regulated metabolites but exclusively in male brain tissue (male control and nKO *n* = 6 and female control and nKO *n* = 5). Steady-state TCA cycle intermediates are labeled in blue, and the most affected are labeled. Included above the volcano plots is a schematic of the metabolic pathways affected by reduced IIS in the brain, highlighting metabolites up-regulated in old Irs1KO only (purple squares), old nKO males only (green squares), and old Irs1KO and nKO males (blue squares). All error bars correspond to SD. Detailed statistical values are found in table S1.

We next asked which molecular mechanisms in the brain might underlie the improved metabolic health of nKO males. Gene expression studies in the liver tissue of Irs1KO mice showed regulation of genes associated with OXPHOS and the tricarboxylic acid (TCA) cycle (*15*), and activation of Forkhead box O1 transcription factor (FOXO1) in the liver resulted in a reduction in the activity of the mitochondrial electron transport chain (*17*), suggesting a change in mitochondrial function upon reduced IIS. Mitochondrial function in neurons is essential for neurotransmission, synaptic maintenance, and calcium homeostasis (*18*). Mitochondrial function has been implicated in the longevity-dependent response to IIS reduction in the brain of *Drosophila* (*51*). However, the effect of reduced IIS on mitochondrial function has not yet been investigated in the mammalian brain. Therefore, we assessed the effect of loss of IRS1 in the brain on OXPHOS by performing high-resolution respirometry on the permeabilized brain tissue of young and old Irs1KO mice. There was no difference in basal respiration in young (fig. S12A) or old (Fig. 4A) Irs1KO mice of either sex, suggesting that the loss of IRS1 function did not affect mitochondrial function in baseline conditions. Next, we determined the mitochondrial spare respiratory capacity, which indicates how much capacity a cell has to deal with acute additional energy demands. Neuronal reduction in spare respiratory capacity has been linked to age-associated neurodegenerative disorders such as Parkinson’s disease (*52*–*54*). Old male Irs1KO mice showed a significantly reduced mitochondrial spare respiratory capacity (Fig. 4C). In contrast, old females or young animals showed no change in mitochondrial spare respiratory capacity (fig. S12B). Next, we measured nicotinamide adenine dinucleotide (NAD^+^) and reduced form of NAD^+^ (NADH) levels, which are some of the redox cofactors required for intraprotein electron transfer in the mitochondria to produce the proton gradient required for oxygen consumption and ATP generation. While total NAD levels were not changed (fig. S13A), we found a significant reduction in NADH levels only in the brain of old Irs1KO males (two-way ANOVA, sex × genotype interaction *P* = 0.0196) (fig. S13C) and consequently an increased ratio of NAD^+^/NADH in male Irs1KO mice (two-way ANOVA, sex × genotype interaction *P* = 0.0530) (fig. S13D). To test whether this decrease in NADH levels might be limiting for mitochondrial respiration (Fig. 4C), we measured respiration in the presence of saturating NADH levels. Under these conditions, respiration of old male Irs1KO brains was not changed (fig. S13), suggesting the limitation of NADH levels as a potential factor contributing to the reduced maximal respiration in the brain of old Irs1KO males.

To address whether this phenotype was caused specifically by the lack of IRS1 in neurons, we also measured spare respiratory capacity in nKO mice. Consistent with the results in Irs1KO mice, nKO mice showed a male-specific reduction in mitochondrial spare respiratory capacity at old age (Fig. 4, B and D), and this was unchanged in Syn1Cre control mice (fig. S14, A and B), indicating that this phenotype was specific to neuronal reduction of IIS. Thus, reduced neuronal IIS lowered mitochondrial spare respiratory capacity in old male Irs1KO and nKO mice, suggesting a low level of mitochondrial stress as basal respiration was unaffected (*55*).

### Lack of IRS1 causes a male-specific induction of the ISR in aged brains

Impaired mitochondrial function has been shown to activate *Atf4* signaling (*20*) and thereby the ISR (*56*, *57*). Moreover, an increase in ATF4 activity has been identified as a common feature of interventions that increase life span in mice (*21*). Therefore, we measured *Atf4* transcript levels by qRT-PCR in the brains of Irs1KO mice, as neuronal ATF4 protein is usually rapidly degraded (*58*). *Atf4* mRNA levels were unchanged in female mice but specifically increased in the brain of old male Irs1KO mice (Fig. 4E and fig. S12C), consistent with the male-specific mitochondrial dysfunction at old age. In line with this finding, *Atf5* expression levels were also only significantly up-regulated in old male Irs1KO mice (Fig. 4E). Expression level of *Chop*, an *Atf4* target gene commonly up-regulated in long-lived mice (*21*), was also increased specifically in male Irs1KO mice (Fig. 4E). One of the main mechanisms for the activation of ATF4 signaling is through EIF2A phosphorylation via EIF2A kinases including PERK, GCN2, PKR, and HRI (*59*). Therefore, we measured EIF2A phosphorylation in brain lysates of Irs1KO mice. However, we did not detect significant changes in the levels of p-EIF2A or the ratio of p-EIF2A to total EIF2A levels (fig. S15, A to C). These results suggested that lack of IRS1 induced ISR in the brains of old males independent of EIF2A. To address whether deletion of IRS1 in neurons was sufficient to activate the ISR, we measured *Atf4*, *Atf5*, and *Chop* expression in the brain of nKO mice. As in Irs1KO mice, no significant change in expression in these genes was observed in young male or in female nKO mice (fig. S12D). However, *Atf5* and *Chop* transcripts were significantly up-regulated in old male nKO mice, and there was a tendency for *Atf4* to be up-regulated (Fig. 4F). Moreover, consistent with the lack of mitochondrial dysfunction in Syn1Cre mice, we did not detect any difference in the expression of the ISR marker genes (fig. S14C). Thus, our data suggest that neuronal loss of IRS1 is sufficient to trigger the ISR specifically in the brain of old male mice.

### Loss of neuronal IRS1 leads to male-specific metabolic adaptations during aging

The ISR pathway has been shown to trigger ATF4-dependent cytoprotective metabolic adaptations (*20*), which are particularly relevant for metabolic rewiring in response to mitochondrial stress (*60*–*62*). Therefore, we used targeted metabolomics on mouse brains to measure metabolite levels that have been associated with the ISR response. NAD^+^ is reduced to NADH as part of the TCA cycle. To address whether this was due to a reduction in TCA cycle progression, we used targeted metabolomics to measure TCA metabolites. However, there was no significant change in the detected TCA metabolite levels between old (Fig. 4, G and H) and young Irs1KO mice and control animals (fig. S12, E and F), suggesting that changes in the TCA do not explain the decreased NADH levels. Methionine, asparagine, and threonine levels were increased specifically in the brain of old male but not female Irs1KO mice (Fig. 4G). These findings are consistent with previous reports that showed a shift in amino acid levels in response to ISR, leading to an accumulation of amino acids such as threonine (*20*), methionine (*63*), glycine, and serine (*64*), potentially due to reduced catabolism of these amino acids. In contrast, these amino acids were not increased in the brain of young Irs1KO males (fig. S12E). Similar to the results observed in Irs1KO mice, threonine, methionine, glycine, and serine accumulated specifically in the brain of old nKO mice (Fig. 4H) but not young nKO mice (fig. S12F), suggesting that these metabolic changes are the result of reduced IIS in neurons. The observed metabolic changes are consistent with the hypothesis that loss of IRS1 in neurons of males during aging triggers the ISR, which then induces a cytoprotective metabolic program.

### IRS1 deletion in peripheral tissues did not induce ISR gene expression

To assess whether deletion of IRS1 in tissues other than neurons also induces the ISR, we measured *Atf4*, *Atf5*, and *Chop* transcript levels in the liver, muscle, and adipose tissue of the respective tissue-specific IRS1 KOs. In contrast to the findings in the nKO males, ISR marker genes were not up-regulated in the liver, muscle, or BAT of old lKO, mKO, or fKO mice, respectively (fig. S16, A to C), suggesting that neuronal tissue is particularly susceptible to IRS1 deletion–mediated ISR. In summary, neuronal deletion of IRS1 caused a male-specific reduction in mitochondrial spare respiratory capacity, activation of *Atf4* signaling, and metabolic adaptations consistent with an activated ISR during aging.

### Neuronal IRS1 deletion induces sex-specific mitochondrial ISR in nonaffected tissues

**Fig. 5.**
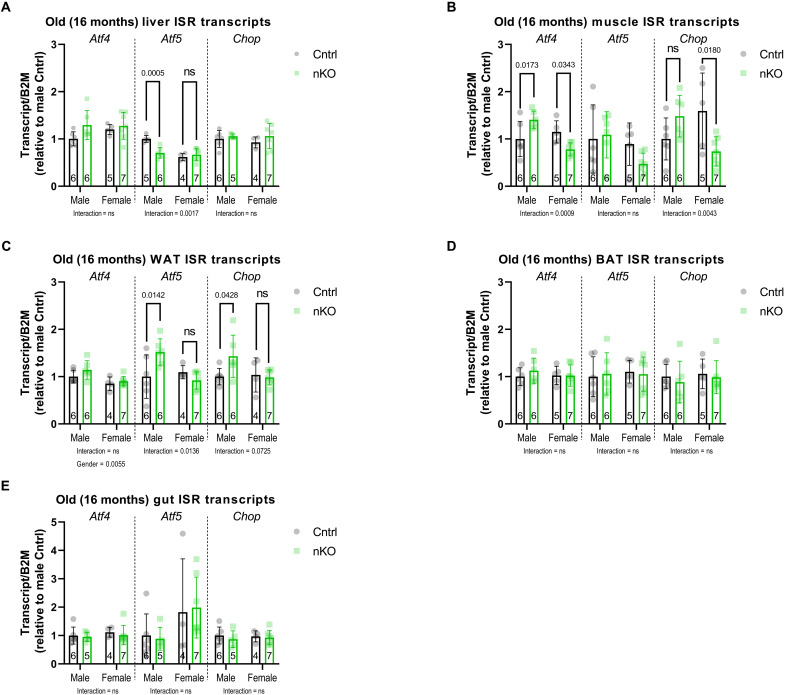
Sex-specific mitochondrial ISR can be activated in nonaffected tissues. qRT-PCR performed on the cortex of nKO mice in old (16-month-old) mice targeting various ISR markers. (**A**) Liver ISR transcripts in nKO mice and their control littermates revealed a sex-specific down-regulation in hepatic *Atf5* levels of male nKO mice. (**B**) Muscle *Atf4* and *Chop* transcripts were significantly up-regulated in nKO males but significantly down-regulated in females, while *Atf5* levels were unchanged. (**C**) WAT *Atf5* transcripts were significantly up-regulated in nKO males in a sex-specific manner, while *Chop* levels showed a trend that did not reach significance. BAT (**D**) and gut (**E**) ISR transcripts were not affected in nKO mice. Detailed statistical values are found in table S1.

Previous studies in *Caenorhabditis elegans* have reported that non–cell-autonomous signals due to electron transport chain disruption in neurons are sufficient to lead to life-span extension by activating mitochondrial stress in the intestine (*65*). Whether mitochondrial ISR induction in one tissue sends stress signals in nonaffected tissues in mammals is still unclear. An in-depth study using a mitochondrial myopathy mouse model characterized the temporal progression and intertissue response to muscle mitochondrial ISR, such as increased brain uptake of glucose, but did not detect up-regulation of mitochondrial ISR in nonaffected tissues (*66*). We asked whether IRS1-mediated ISR activation in the brain can activate a mitochondrial ISR signal in peripheral tissues. We measured ISR marker transcripts in the liver, muscle, WAT, BAT, and intestine (Fig. 5, A to E) in old male and female nKO mice. We detected a sexually dimorphic *Atf4* and *Chop* signal in the muscle, where transcripts were significantly up-regulated in nKO males but down-regulated in females (Fig. 5B). Moreover, we found that WAT of nKO males presented with a sex-specific up-regulation of *Atf5* and *Chop* levels (Fig. 5C). However, liver, BAT, and gut ISR markers were not significantly changed (Fig. 5, A, D, and E). These data suggest that the brain may be secreting a factor or involved in a circuit that is initiating mitochondrial ISR in some peripheral tissues. We tested whether IRS1 deletion in the liver, muscle, and fat tissue–specific Irs1KO mice led to an up-regulation of ISR markers in a nonaffected tissue such as the brain (fig. S17), but we did not detect any evidence of ISR activation.

### Neuronal IRS1 deletion induces systemic benefits through an FGF21-independent mechanism

Previous reports studying mitochondrial dysfunction in the central nervous system (CNS) found evidence of systemic responses such as improved glucose metabolism through improved glucose uptake in nonaffected organs (*67*, *68*). We hypothesized that fibroblast growth factor 21 (FGF21) was a likely candidate in IRS1 deletion–mediated health benefits, given the previous reports linking FGF21 to increased EE (*69*) and insulin sensitivity (*70*), as well as being responsive to mitochondrial stress (*71*). Moreover, the role of the mitokine, *Fgf21*, in the CNS ISR response has been described in the hypothalamus (*67*, *68*). However, we tested for up-regulation of *Fgf21* transcripts and FGF21 protein levels in the cortex of old nKO and Irs1KO mice, the same region where we detected mitochondrial dysfunction. We did not find detectable levels in brain cortical tissue as previously reported (*66*), which seems to suggest that there could be region specificity to *Fgf21* expression in the brain. The liver is a primary source of circulating FGF21 (*72*). Moreover, reports have linked male-specific life-span (*73*) and health-span improvement (*74*) with a hepatic *Fgf21* signal (*69*). To investigate whether IRS1 deletion led to an up-regulation of hepatic *Fgf21*, we first tested for the activation of ISR in the liver. We found a male-specific up-regulation of *Atf4* transcripts in livers of old Irs1KO mice (two-way ANOVA, sex × genotype interaction *F*_1,20_ = 5.464, *P* < 0.0299; fig. S18A) and a trend of up-regulation in *Atf5* levels. Moreover, we also detected a genotype-dependent up-regulation of *Chop* in both old male and female Irs1KOs. Next, we assessed levels of p-EIF2A to determine whether the canonical ISR pathway was being activated in the old Irs1KO livers. Unexpectedly, we detected a sex-specific reduction of p-EIF2A in old males (fig. S18B). This was not due to a change in total EIF2A levels (fig. S18C) but was also present when p-EIF2A was normalized to total levels (fig. S18D). Moreover, when we measured hepatic *Fgf21* transcript levels, we detected a significant reduction of levels in the Irs1KO livers (fig. S18E). We also confirmed that *Fgf21* transcript levels in nKO mice were not significantly different (fig. S18F), consistent with our results from the hepatic ISR (Fig. 5A). While *Fgf21* is downstream of *Atf4* (*75*), our data suggest that the *Atf4* regulation of *Fgf21* is context specific. This down-regulation of hepatic *Fgf21* in our Irs1KO mice is consistent with a previous report of IRS1 and IRS2 double-KO mice, where there was a reduction in hepatic *Fgf21* expression in mutant mice during fasted and refed conditions (*42*).

Given that other organs are also implicated in FGF21 secretion (*76*), we conducted an FGF21 enzyme-linked immunosorbent assay (ELISA) on plasma samples from old male and female Irs1KO mice to assess whether a systemic ISR signature was being transmitted through FGF21 levels from other tissues. Consistent with the data of *Fgf21* transcripts from the liver of Irs1KO mice (fig. S18E), we found a significantly reduced level of circulating FGF21 in the plasma of old Irs1KO mice (fig. S18G). A reason could be an increase in nuclear FOXO1 due to the reduction in IIS, as hepatic FOXO1 has been implicated in reducing circulating FGF21 levels (*77*). Therefore, our data suggest the existence of a mechanism by which neuronal ISR induces a systemic response independent of FGF21.

## DISCUSSION

Our study provides a systematic study into the tissue-specific role of IRS1 deletion on longevity. We find that IRS1 deletion is a robust intervention capable of longevity induction in different mouse strains, suggesting that mechanisms leading to longevity are independent of genetic background and are broadly relevant to the aging process. Tissue-specific IRS1 deletion in major insulin-sensitive organs was insufficient to extend life span. Specific IRS1 deletion in the liver, muscle, and fat tissue did not improve health at old age in male or female mice, whereas neuron-specific IRS1 deletion led to male-specific increase in health outcomes that recapitulate phenotypes of Irs1KO mice (fig. S19). These findings highlight the importance of neuronal IIS and sex in modulating health outcomes in mice. Furthermore, we found that reduced neuronal IIS led to male-specific mitochondrial dysfunction, activation of *Atf4* signaling, and metabolic adaptations consistent with an ISR response at old age. We find evidence for mammalian mitochondrial ISR spread from the brain to peripheral organs through a sex-specific, FGF21-independent mechanism in old mice (fig. S20).

We used a robust C3B6F1 hybrid mouse strain to mitigate confounds of inbred lines on complex phenotypes such as longevity (*78*). There was no effect on hybrid Irs1KO viability, and progeny were born in a Mendelian ratio. We replicated previous reports of Irs1KO mice leading to life-span extension and improved health parameters in a new robust hybrid background. Previous reports have found that the mouse genetic background affects the extent of life-span extension in dietary, genetic, or pharmacological longevity interventions (*79*). Initially, we generated a global IRS1 deletion model under the ubiquitous actin-Cre driver. However, there was a depletion of *Actin Cre+/T::Irs1fl/fl* pups generated from *Actin Cre+/+::Irs1fl/+* and *Actin Cre+/T::Irs1fl/fl* matings, suggesting reduced viability of IRS1 deletion mice. We also could not successfully breed C57BL/6N Irs1KO mice, because Irs1KO progeny dropped in the third generation of backcrossing to approximately 4%. The inability to generate this mutant line could be due to an interaction effect between the IIS intervention and the C57BL/6 background. Haploinsufficiency in the IGF1R gene in the 129Sv mouse background led to a significant life-span extension in females (33%) but only a trend in males (16%) (*80*). In contrast, Bokov and colleagues (*81*) repeated the experiment in a C57BL/6 background and found only a modest life-span extension in females (5%), and males showed a small but insignificant reduction. When Bokov and colleagues (*81*) tried the same intervention in the hybrid 129SBL6F1 background, no life-span extension was detected in males or females. These findings suggest that reducing IIS in the C57BL/6 background may lead to a strain-specific sexual dimorphism leading to benefits in females but adverse effects in males. These two studies highlight the importance of replication and impact of inbred mouse strains on aging studies and the effect of sex on reduced IIS interventions. Together, these reports fill us with confidence that the effect of IRS1 deletion on longevity is robust and is targeting biological mechanisms of aging contrary to rescuing a deficit in an inbred mouse line.

The tissue-specific role of reduced IIS has been investigated extensively in the context of metabolic disorders to study the consequences of tissue-specific insulin resistance (*27*), but the effect of tissue-specific IIS disruption on life span is unknown. We measured the life span and health span of life-long and tissue-specific IIS reduction by deletion of IRS1 in the liver, muscle, fat, and brain of male and female mice. We found no evidence for significant extension of life span. Conversely, in *Drosophila*, the causal mechanisms that contribute to extended life span are tissue specific (*51*), potentially due to the lack of paralogs that could rescue pathway activity. Global reduction of IIS by deletion of IIS proteins upstream in the pathway, such as IR, IGF1R, or IGF1, leads to early mortality (*82*, *83*). However, partial disruption, either by targeting specific tissues such as fat (*39*) or brain tissue (*84*) or proteins that do not abrogate the pathway (*7*), can lead to life-span extension. Recent reports of ectopic recombination in the CNS and in peripheral metabolic organs may confound the fat-specific (*85*) and brain-specific (*46*) findings, respectively. When IR was deleted in all peripheral tissues after adulthood, no life-span extension was observed, but a large and significant reduction in male life span was detected (*86*). This finding again not only highlights the sensitivity of C57BL/6 males to reduced IIS but also suggests that IIS reduction during development plays a role in life-span modulation. Therefore, we built on previous approaches by using a constitutively active but partial IIS disruption, by deleting the IRS1 protein, in tissue-specific models with a proven record of specific recombination and confirmed by qRT-PCR. Our findings suggest that either a tissue not tested in our study or multiple tissues are necessary for tissue-specific IRS1 deletion–mediated life-span extension.

Female Irs1KO mice, which had reduced glucose tolerance and insulin sensitivity, were longer-lived than control animals, suggesting that insulin resistance does not necessarily limit survival. In line with the hypothesis that insulin resistance and longevity are not necessarily coupled, Irs1KO mutant males showed a similar life-span extension to females but showed improved insulin sensitivity, and nKO male mice showed improved insulin sensitivity but were not long-lived. Furthermore, male lKO and fKO mice were not long-lived but showed a trend toward increased glucose tolerance. In contrast to deletion of IRS1, loss of RICTOR, a component of the mTORC2 complex, leads to a sex-specific decrease in male life span even when the intervention is induced in adulthood (*87*). The short life span of these animals has at least in part been attributed to their insulin resistance. Fat-specific RICTOR deletion leads to insulin resistance as the mice display elevated levels of circulating insulin but reduced insulin sensitivity at young age (*88*). Moreover, RICTOR deletion under the Nestin promoter leads to severe reduction in neuronal and overall brain size (*89*). In addition, hypothalamic deletion of RICTOR leads to reduced activity, survival, glucose tolerance, and insulin sensitivity in male and female mice (*90*). We observed the opposite effects in activity and insulin sensitivity in male nKO mice. Furthermore, we observed no difference in brain size, survival, or glucose tolerance in neither male nor female nKO mice. Thus, interventions into different branches of the IIS/mTOR network cause specific effects on metabolic health and survival. While our results do not exclude that insulin resistance is a driver of the metabolic dysfunction in RICTOR mutant animals, they show that, in mice, insulin resistance is not necessarily coupled with a short life.

We found that the deletion of IRS1 specifically in neurons led to improved systemic metabolic changes at old age, without detrimental effects on body size and viability observed in global C57BL/6J Irs1KO mice. Neuronal IIS reduction led to enhanced insulin sensitivity, increased EE, and improved motor activity, specifically in old male mice. However, these changes were insufficient to extend life span. In mammalian models of neurodegenerative disease, manipulation of IIS in the CNS improves cellular resilience, especially with regard to proteotoxic stress (*91*). The role of IIS in the CNS in physiological aging is still controversial in mammals (*92*). In *C. elegans*, neuronal IIS reduction can induce longevity by increasing the resistance of neurons to free radicals (*93*) or by maintenance of mitochondrial integrity through non–cell-autonomous mechanisms. The health-span benefits that we observed in nKO mice are consistent with data obtained from fly models (*94*, *95*). Moreover, similar changes in insulin sensitivity and locomotion were obtained from a study where IR was deleted only in male mice and specifically in the hypothalamus under high-fat diet conditions (*50*). One possibility for the sex difference is the age-associated and tissue-specific difference in activation of the IIS pathway in male and female C57BL/6 mice (*96*). However, given the interaction of mouse background and IIS, this needs to be tested further in studies that include both male and female mice in different strains. Another possibility could be due to differences in X chromosome inactivation (Xi). Xi is when one of the two X chromosomes in females is inactivated to balance the expression of genes on the X chromosome between XX females and XY males. The process of Xi is incomplete and can lead to some genes escaping the inactivation in a tissue-specific manner and end up regulating somatic genes (*97*). Moreover, different cell types in the brain have varying degrees of Xi with potential effects on neuronal development and function (*98*). More sex comparative studies need to be conducted to understand how IIS or ISR genes in the brain are being regulated by genes escaping Xi.

Previous studies found a reduction in hepatic mitochondrial function in response to IIS reduction but did not disclose the sex of mice used (*17*). We show here that IRS1 deletion reduced mitochondrial spare respiratory capacity and increased the NAD^+^/NADH ratio in the brain. In contrast to our finding, NAD^+^/NADH ratio was reduced in the liver of IRS1 and IRS2 double-KO mice (*17*). This might be due to differences between the liver and brain tissue in response to IRS1 deficiency or due to the stronger down-regulation of pathway activity in the double mutant animals. Accordingly, while lack of IRS1 only affected maximum respiration, lack of IRS1 and IRS2 affected both basal and maximum respiration (*17*). The finding that maximum respiration was not negatively affected in old *Irs1* mutant males when NADH was externally supplied in saturating levels suggests that NADH levels may contribute to a bottleneck for mitochondrial spare capacity in the male insulin mutant brain.

The reduction in mitochondrial capacity without a change in basal mitochondrial respiration could suggest that physiological neuronal function is not impaired, but mitochondria are stressed (*55*). The activation of mitochondrial ISR is a sign of a cellular response to stress to promote resilience (*20*). Activation of the ISR involves phosphorylation of EIF2A and a subsequent increase in ATF4 protein levels (*59*). In contrast, we only detected a small trend for increased EIF2A phosphorylation, suggesting that EIF2A phosphorylation might not be the main driver for IRS1-dependent ISR. Consistently, recent reports have described an independent mechanism of *Atf4* activation through mitochondrial dysfunction (*20*). Whether this also occurs in the brain and whether sex differences exist remain to be elucidated. Previous reports investigated the effects of brain mitochondrial stress only in male mice and found it sufficient to induce a systemic endocrine signal (*68*), but the role of sex and the effect of brain mitochondrial ISR on metabolic health and aging remain to be elucidated. A landmark study on the effect of long-term systemic ISR in the muscle of male mice found that the first stages of muscle-initiated ISR are defined by the secretion of FGF21 and growth differentiation factor 15 mitokines (*66*). Moreover, they found systemic consequences in organs not affected by ISR, such as the brain, where an increase in glucose uptake was observed. Previous reports that investigated the loss of male brain mitochondrial integrity also found evidence of a systemic signal by the induction of FGF21 (*67*, *68*). However, FGF21 expression was not induced in the brain or liver of Irs1KO mice, and FGF21 plasma levels were strongly reduced in long-lived Irs1KO mice. Thus, it is unlikely that FGF21 constitutes a brain-derived signal downstream of IRS1. Consistently, with this hypothesis, brain-derived FGF21 has been shown to directly affect brain function but not peripheral metabolism (*99*). Furthermore, FGF21 signaling is not required for mitochondrial ISR in cardiac tissue, suggesting that activation of FGF21 can be context specific (*100*). Moreover, in the absence of *Atf4* signaling, amino acid restriction can still lead to the up-regulation of *Fgf21* signaling, demonstrating that *Atf4* and *Fgf21* signaling are not always coupled (*101*). Another potential mechanism leading to systemic effects in nonaffected tissues of nKO mice could be the presence of neural circuits innervating specific peripheral organs. Although we observed similar systemic metabolic phenotypes in female Irs1KO mice, we could not detect mitochondrial dysfunction or a similar ISR response in female brain tissue. This suggests that the female phenotypes may be mediated through a different mechanism or that the ISR in females functions in a different way. A previous report of mitochondrial DNA damage–induced mitochondrial stress in muscle tissue revealed sex differences in muscle-free amino acids in aged mice (*102*), but sex differences in other parameters were not assessed. Mammalian models of nutrient stress–induced systemic ISR implicate an endocrine signal leading to a male-specific life-span (*73*) and health-span (*74*) improvement, providing some evidence of sex specificity in this pathway. Unfortunately, most previous studies in the ISR field only studied males, but our report suggests that sex as a variable should be considered in future studies.

### Limitations

We acknowledge several limitations in this study. First, given that all our interventions were constitutive, we cannot be certain to what extent the developmental effect of reduced IIS contributes to the observed phenotypes. Although we could not detect changes in EE, locomotor activity, ISR transcripts, or mitochondrial function at young age, we cannot rule out lingering developmental effects later in life. Time-specific genetic tools could target the IIS intervention to adulthood and help eliminate any developmental confounds. Second, EE in Irs1KO mice is confounded by their reduced body size. The increased EE could be partly attributable to the body size difference, even with normalization to lean mass or body mass, because we cannot adjust for size by analysis of covariance (ANCOVA) because the difference in the range of values between wild-type and mutant mice is too large. However, the finding that nKO mice, where we could adjust the data using ANCOVA, showed a similar phenotype might suggest that this phenotype is not simply caused by differences in body weight. Last, we detected a significant improvement in insulin sensitivity of young male Irs1KO mice, whereas a previous hyperinsulinemic-euglycemic clamp study found evidence of insulin resistance in the muscle of Irs1KO mice (*36*). The clamp used is a more robust method for measuring insulin sensitivity as a distinction between liver, fat, and muscle insulin resistance can be made. One potential explanation of this discrepancy could be the difference in mouse background and age in the two studies. The study (*36*) did not report the mouse strain, but they did mention that the male Irs1KO mice used were 6 weeks old in contrast to males in this study that were 6 months old. The young wild-type hybrid C3B6F1 male mice did not respond to insulin at 6 months of age, which may have exacerbated the difference between young Irs1KO and their littermates (fig. S1E). However, young control C57BL/6N male mice responded to intraperitoneal insulin injection at 3 months of age (fig. S7), potentially highlighting the role of mouse background and age in this metabolic outcome. Another limitation in this study is our inability to detect the ATF4 protein levels in brain tissue. This might be due to its transient nature in the unstressed state (*58*). Consistently, C/EBP homology protein (CHOP) and ATF4 levels were undetectable in naïve brains, and only under strong stress conditions, such as severe endoplasmic reticulum stress, were levels chronically and strongly up-regulated (*103*). Therefore, the reason that we did not detect ATF4 or CHOP protein levels in the brain of Irs1KO mice might indicate a mild induction of the ISR in our model, consistent with the mild mitochondrial respiration defect and transcriptional up-regulation of *Atf4*. Last, we only measured steady-state levels of metabolites in the brains of Irs1KO and nKO mice, which do not allow us to address metabolic flux. Thus, metabolite tracing experiments would be necessary to test whether, e.g., changes in TCA flux are contributing to the reduced NADH levels in the brain of Irs1KO males.

In conclusion, we find evidence for a unique and causal role of neuronal IRS1 deletion in triggering systemic health benefits in males that recapitulate those found in Irs1KO mice in a sex-specific manner. Moreover, these data suggest that males and females respond differently to ISR in response to IRS1 deletion, and evidence for sex-specific ISR activation due to nutrient stress has been reported previously (*74*). However, it is currently unclear to what extent is ISR sex specific in mammals and how different tissues react to the induction of ISR. Together, our study reveals details of how reduced IIS triggers a global response across the organism, some of which are tissue specific and some are sex specific, reflecting a combination of local and systemic cues. Moreover, the newly identified sex-specific mechanisms in response to reduced neuronal IIS provide potential avenues for therapeutic interventions. However, some tissues were not included in this study, and they may respond differently with different outcomes for the whole organism. We suggest that future research should investigate both sexes when studying the ISR pathway in response to various stressors and in a tissue-specific manner.

## MATERIALS AND METHODS

### Mouse experiments and animal care

#### Mouse generation

Irs1KO mice were generated previously (*15*) and provided by D.J.W. Previous data of Irs1KO mice were generated in the C57BL/6J background (*15*). However, because of genetic mutations in the C57BL/6J line that may confound metabolic traits, we chose to generate the Irs1KO mice in the C57BL/6N background (*104*). After embryo transfer of Irs1KO embryos from C57BL/6J into C57BL/6N mice, we observed Irs1KO progeny to drop markedly in the third generation to approximately 1% Irs1KO mice being born. Following attempts to optimize diet and housing conditions, Irs1KO progeny continued to stay far below the expected ratio in subsequent generations (~7% Irs1KO mice with normal levels of heterozygous and wild-type mice observed in litters). In addition, we tried to generate a Cre-specific whole-body Irs1KO in the C57BL/6 background via the actin-Cre driver line. However, attempts to generate *Actin Cre+/T::Irs1fl/fl* led to similarly low rates of Irs1KO mice (~2%). Therefore, we generated the Irs1KO mouse in a more robust C3B6 hybrid mouse background (*78*). *Irs1*KO mice were backcrossed for four generations into the C57BL/6N (C57BL/6NCrl, Charles River Laboratories) and C3H/HeOuJ (RRID: IMSR_JAX:000635, The Jackson Laboratory) background. Heterozygous C3H/HeOuJ *Irs1*^−/+^ females were mated with heterozygous C57BL/6N *Irs1*^−/+^ males to generate hybrid C3B6F1 whole-body Irs1KO mice.

*Irs1loxP* mice were generated as previously described (*29*). For tissue-specific KO of *Irs1*, *Irs1loxP/loxP* mice were crossed with mice expressing Cre-recombinase under the control of the mouse albumin enhancer and promoter and the mouse alpha-fetoprotein enhancers [*AlfpCre* mice; (*30*)], the control of the creatine kinase promoter [CkmmCre mice; (*31*)], the control of the adiponectin promoter [AdipoqCre; (*32*)], the control of the villin promoter [Villin1Cre mice; (*33*)], or the control of the rat synapsin I promoter [*Syn1Cre* mice; (*34*)]. Breeding *Irs1loxP/loxP AlfpCre* mice with *Irs1loxP/loxP* mice resulted in hepatocyte-specific *Irs1* deletion (*AlfpCre::Irs1fl/fl* denoted as lKO) and littermate control (*Irsfl/fl*) mice. Breeding *Irs1loxP/loxP CkmmCre* mice with *Irs1loxP/loxP* mice resulted in skeletal muscle–specific *Irs1* deletion with partial deletion in cardiac muscle (*CkmmCre::Irs1fl/fl* denoted as mKO) and littermate control (*Irsfl/fl*) mice. Breeding *Irs1loxP/loxP AdipoqCre* mice with *Irs1loxP/loxP* mice resulted in fat-specific *Irs1* deletion in both WAT and BAT (*AdipoqCre::Irs1fl/fl* denoted as fKO) and littermate control (*Irsfl/fl*) mice. To avoid germline deletion in Syn1Cre in mice (*105*), only female *Irs1loxP/loxP* Syn1Cre mice were bred with male *Irs1loxP/loxP* mice to produce neuron-specific *Irs1* deletion (*Syn1Cre::Irsfl/fl* denoted as nKO) and littermate control (*Irsfl/fl*) mice.

#### Mouse husbandry

All mice were maintained in groups of four to five same-sex (only females were randomized) individuals under specific pathogen–free conditions in individually ventilated cages (Techniplast UK Ltd., Kettering, Northamptonshire, UK) to provide a controlled temperature and humidity environment with 12-hour light/12-hour dark cycle (lights on from 0600 to 1800) and provided ad libitum access to food [ssniff R/M-H phytoestrogen-poor (9% fat, 34% protein, and 57% carbohydrate), ssniff Spezialdiäten GmbH, Soest, Germany] and sterile-filtered water. Sentinel mice in the animal room were regularly checked to be negative for mouse pathogens according to the Federation of the European Laboratory Animal Science Association (FELASA) recommendations.

#### Mouse ethics

Mouse experiments were performed in accordance with the recommendations and guidelines of the FELASA, with all protocols approved by the Landesamt fur Natur, Umwelt und Verbraucherschutz Nordrhein-Westfalen, Germany. Ethical permission requests were filed under 84-02.04.2014.A424 and 81-02.04.2019.A078.

#### Mouse genotyping

Mutant mice were identified by PCR genotyping using DNA extracted from ear clip biopsy and amplified using GoTaq G2 DNA Polymerase; moreover, tail clips were taken from mice at death for genotype confirmation. Primers used to genotype as well as the expected size of amplicons, of *Irs1* wild type, Irs1KO, *Irs1 LoxP* floxed allele, and different *Cre* lines are listed in table S1.

#### Mouse tissue collection

Mice were euthanized by transcardial perfusion with phosphate-buffered saline + EDTA (only for Irs1KO and nKO mice) after general anesthesia with a cocktail of ketamine (120 mg/kg) and xylazine (10 mg/kg) with supplementary isoflurane (5%) until no reflex response was observed. Then, blood was collected by cardiac puncture in tubes with EDTA; plasma-EDTA was isolated by centrifugation at 1000*g* for at least 10 min at 4°C before aliquoting and storage at −80°C. Mice were rapidly decapitated, and then the skull and body were dissected by two different scientists simultaneously to minimize tissue deterioration. The brain was removed from the skull, and different brain regions were quickly isolated and snap-frozen in liquid nitrogen. The same cortical brain region was dissected for mitochondrial respirometry and prepared separately. The body of the animal was dissected and organs were collected for histology in paraformaldehyde or snap-frozen in liquid nitrogen for molecular analysis. The same procedure was performed for lKO, mKO, and fKO with the exception of perfusion. Mice from lKO, mKO, and fKO lines were euthanized by cervical dislocation, followed by rapid tissue removal as mentioned above.

### Mouse metabolic phenotyping

A longitudinal cohort of mice was assessed at young (Irs1KO = 6 months, while lKO, mKO, fKO, and nKO = 4 months) and old (Irs1KO and lKO, mKO, fKO, and nKO = 16 months) age for general metabolic health outcomes.

#### Body composition

Body fat and lean mass content were measured in vivo by nuclear magnetic resonance using a minispec LF50H (Bruker Optics).

#### Collection of blood samples and determination of blood glucose levels

A small drop of blood was obtained from the tail of mice. Blood glucose levels were determined using an automatic glucose monitor (Accu-Check Aviva, Roche). Determination of blood glucose and collection of blood samples were always performed in the morning to avoid deviations due to circadian variations.

#### Insulin tolerance test

After determination of basal blood glucose levels, each animal received an intraperitoneal injection of insulin (0.75 U/kg of body weight; Sanofi). Blood glucose levels were measured 15, 30, and 60 min after insulin injection.

#### Glucose tolerance test

Glucose tolerance tests were performed in the morning with animals after a 16-hour fast. After determination of fasted blood glucose levels, each animal received an intraperitoneal injection of 20% (w/v) glucose (10 ml/kg of body weight). Blood glucose levels were measured 15, 30, 60, and 120 min after the glucose injection.

#### Indirect calorimetry

Indirect calorimetry, locomotor activity, drinking, and feeding were monitored for singly housed mice in purpose-built cages (Phenomaster, TSE Systems). Parameters such as food consumption, respiration, and locomotor activity were measured continuously for 48 hours after 1 day of acclimatization and 2 days of training in similar cages. Values for locomotor activity were averaged for active and inactive phases separately for the 48-hour duration with the exception of the first and last hour of each phase. Metabolic rate was assessed by regression analysis using body weight as a covariate as recommended (*106*), except for Irs1KO mice due to the effect of the mutation on body size.

### Metabolomics

#### Two-phase metabolite extraction of polar and lipophilic metabolites of brain tissue

For the preparation of polar and lipophilic metabolites, between 10 and 30 mg of snap-frozen mouse tissue (Irs1KO young = 5 months and old = 19 months; nKO young = 5 months and old = 22 months) was collected in 2-ml Eppendorf (www.eppendorf.com) tubes. For the extraction of the snap-frozen material, the tissue was homogenized to a fine powder using a ball mill–type grinder (TissueLyser II, Qiagen, 85300). For the homogenization of the frozen material, one liquid nitrogen–cooled 5-mm stainless steel metal ball was added to each Eppendorf tube, and the frozen material was disintegrated for 1 min at 25 Hz.

Metabolites were extracted by adding 1 ml of precooled (−20°C) extraction buffer [methyl *tert*-butyl ether (MTBE):ultraperformance liquid chromatography (UPLC)–grade methanol:UPLC-grade water 5:3:2 (v/v/v)], containing an equivalent 0.2 μl of EquiSplash Lipidomix (www.avantilipids.com) as an internal standard. The tubes were immediately vortexed until the sample was well resuspended in the extraction buffer. The homogenized samples were incubated on a cooled (4°C) orbital mixer at 1500 rpm for 30 min. After this step, the metal ball was removed using a magnet, and the samples were centrifuged for 10 min at 21,100*g* in a cooled tabletop centrifuge (4°C). The supernatant was transferred to a fresh 2-ml Eppendorf tube, and 250 μl of MTBE and 150 μl of UPLC-grade water were added to each sample. The tubes were immediately vortexed before incubating them for an additional 10 min on a cooled (15°C) orbital mixer at 1500 rpm, before centrifuging them for 10 min at 15°C and 16,000*g*. After the centrifugation, the tubes contained two distinct phases. The upper MTBE phase contains the lipids, while the lower methanol-water phase contains the polar and semipolar metabolites.

For the lipidomic analysis, 600 μl of the upper lipid phase was collected into fresh 1.5-ml Eppendorf tubes, which were stored at −80°C, until mass spectrometric analysis. The remaining polar phase (~800 μl) was immediately dried in a SpeedVac concentrator and stored dry at −80°C until mass spectrometric analysis.

#### Semitargeted LC-HRMS analysis of amine-containing metabolites of brain tissue

The liquid chromatography–high-resolution mass spectrometry–based (LC-HRMS) analysis of amine-containing compounds was performed using an adapted benzoyl chloride–based derivatization method (*107*). Briefly, the polar fraction of the metabolite extract was resuspended in 200 μl of LC-MS–grade water (Optima-Grade, Thermo Fisher Scientific) and incubated at 4°C for 15 min on a thermomixer. The resuspended extract was centrifuged for 5 min at 21,100*g* at 4°C, and 50 μl of the cleared supernatant was mixed in an autosampler vial with a 200-μl glass insert (Chromatography Accessories Trott, Germany). The aqueous extract was mixed with 25 μl of 100 mM sodium carbonate (Sigma-Aldrich), followed by the addition of 25 μl of 2% (v/v) benzoyl chloride (Sigma-Aldrich) in acetonitrile (Optima-Grade, Thermo Fisher Scientific). Samples were vortexed and kept at 20°C until analysis. For the LC-HRMS analysis, 1 μl of the derivatized sample was injected onto a 100 mm × 2.1 mm HSS T3 UPLC column (Waters). The flow rate was set to 400 μl/min using a binary buffer system consisting of buffer A [10 mM ammonium formate (Sigma-Aldrich) and 0.15% (v/v) formic acid (Sigma-Aldrich) in LC-MS–grade water (Optima-Grade, Thermo Fisher Scientific)]. Buffer B consisted solely of acetonitrile (Optima-Grade, Thermo Fisher Scientific).

The mass spectrometer (Orbitrap ID-X, Thermo Fisher Scientific) was operated in a positive ionization mode recording the mass range mass/charge ratio of 100 to 1000. The heated electrospray ionization source settings of the mass spectrometer were as follows: spray voltage of 3.5 kV, capillary temperature of 300°C, sheath gas flow of 60 arbitrary units (AU), auxiliary gas flow of 20 AU at a temperature of 340°C, and the sweep gas to 2 AU. The RF lens was set to a value of 60%. Semitargeted data analysis for the samples was performed using the TraceFinder software (version 4.1, Thermo Fisher Scientific). The identity of each compound was validated by authentic reference compounds, which were run before and after every sequence. Peak areas of [M + nBz + H]^+^ ions were extracted using a mass accuracy of <5 parts per million (ppm) and a retention time tolerance of <0.05 min. Areas of the cellular pool sizes were normalized to the internal standards [U-^15^N;U-^13^C amino acid mix (MSK-A2-1.2), Cambridge Isotope Laboratories], which were added to the extraction buffer, followed by normalization to the fresh weight of the analyzed sample.

#### AEX-MS for the analysis of anionic metabolites of brain tissue

Extracted metabolites were resuspended in 150 to 200 μl of Optima UPLC/MS-grade water (Thermo Fisher Scientific). After a 15-min incubation on a thermomixer at 4°C and a 5-min centrifugation at 21,100*g* at 4°C, 100 μl of the cleared supernatant was transferred to polypropylene autosampler vials (Chromatography Accessories Trott, Germany) before anion-exchange chromatography mass spectrometry (AEX-MS) analysis. The samples were analyzed using a Dionex ion chromatography system (Integrion, Thermo Fisher Scientific) as described previously (*108*). The detailed quantitative and qualitative transitions and electronic settings for the analyzed metabolites are summarized in table S2. For data analysis, the area of the deprotonated [M − H^+^]^−^ monoisotopic mass peak of each compound was extracted and integrated using a mass accuracy of <5 ppm and a retention time tolerance of <0.1 min as compared to the independently measured reference compounds. Areas of the cellular pool sizes were normalized to the internal standards (citric acid D4), which were added to the extraction buffer, followed by normalization to the fresh weight of the analyzed sample.

### Quantitative real-time polymerase chain reaction

RNA was extracted according to the manufacturer’s instructions using the TRIzol reagent (Thermo Fisher Scientific, 15596018) in Lysing Matrix D tubes (speed 6 for 40 s) (MP Biomedicals, 6913-500). RNA was precipitated with the aid of GlycoBlue Coprecipitant (Thermo Fisher Scientific, AM9515) overnight at −80°C. RNA was treated with deoxyribonuclease using a DNA-free kit (Thermo Fisher Scientific, AM1906) according to the manufacturer’s instructions. Complementary DNA (cDNA) was prepared with the SuperScript III First-Strand Synthesis SuperMix (Thermo Fisher Scientific, 18080400) for qRT-PCR. Samples of cDNA mixed with the PowerUp SYBR Green Master Mix (Thermo Fisher Scientific, 4368706) and primers were validated using a standard curve and loaded in technical quadruplicates for qRT-PCR on a QuantStudio 6 Flex Real-Time PCR System (Thermo Fisher Scientific, 4485691). The ΔΔCt method was used to provide gene expression values after normalizing to the known reference gene *B2M*. Primer sequences used for qRT-PCR are shown in table S3. Samples of cDNA mixed with the TaqMan Fast Advanced Master Mix (Thermo Fisher Scientific, 4444557) and TaqMan Assay probes were loaded in technical quadruplicates for qRT-PCR on a QuantStudio 6 Flex Real-Time PCR System (Thermo Fisher Scientific, 4485691). The ΔΔCt method was used to provide gene expression values after normalizing to the known reference gene *B2M*. Probe catalog numbers used for qRT-PCR are shown in table S3.

### Immunoblotting

Organs immediately frozen in liquid nitrogen were mechanically pulverized using a sterile metal bead in the TissueLyser II (Qiagen, 85300). Protein from samples were extracted using radioimmunoprecipitation assay lysis and extraction buffer (Thermo Fisher Scientific, 89900) supplemented with cOmplete protease inhibitor cocktail without EDTA (Merck, 11836145001) and PhosSTOP phosphatase inhibitor cocktail (Merck, 04906837001); samples were briefly sonicated (10 s, 1-s on and 1-s off, at 20% amplitude; Sonics & Materials Inc., VCX 130) before centrifugation at 13,000*g* for 10 min at 4°C. Proteins were quantified using the Pierce BCA Protein Assay Kit (Thermo Fisher Scientific, 23225), and 15 to 30 mg of protein, depending on the tissue, were loaded on AnykD Criterion TGX Stain-Free Protein Gel (Bio-Rad, 5678125). Proteins were transferred to 0.2-mm Amersham Protran nitrocellulose membranes (GE Healthcare, 10600001) using wet transfer for 45 min at 100 V. Unspecific binding was blocked using 5% nonfat dried milk powder (Labsense, A08301000) in TBST.

Primary antibodies were diluted (see table S3) and incubated with the membrane overnight at 4°C. Horseradish peroxidase–coupled secondary antibodies (1:10,000; Thermo Fisher Scientific) were used according to the primary antibody. Signal was developed using ECL Select (Merck, GERPN2235) or ECL Prime (Merck, GERPN2236) Western Blotting Detection Reagent on the ChemiDoc Imager (Bio-Rad). To allow robust quantification, exposure time of the blots was adjusted to the expression level of each protein individually. Band intensities were quantified using Bio-Rad software. Vinculin was used as a normalization control. Antibodies used in this study are shown in table S3.

### Enzyme-linked immunosorbent assay

FGF21-targeted ELISA was performed on diluted mouse EDTA-plasma according to the instructions of the manufacturer (R&D Systems, MF2100). Briefly, 60 μl of plasma was diluted (1:1) with the provided diluent. All samples, controls, and standards were loaded in technical duplicates.

### Quantification of NAD/NADH

NAD^+^ and reduced NADH were quantified using a colorimetric assay on mouse brain cortical tissue with a slight adjustment to the manufacturer’s instructions (Sigma-Aldrich, MAK037). Cortical samples (~10 mg) were placed in 400 μl of extraction buffer and homogenized with a pedestal (VWR, 431-0098) and then centrifuged at 14,000*g* for 5 min. Supernatant was transferred to 10-kDa filter tubes (Abcam, ab93349) and centrifuged for 10 min. The remaining protocol was performed according to the manufacturer’s instructions with 5 μl of sample loaded per well for the final reaction to measure NAD total and NADH separately (each in duplicate). Analysis was done according to the manufacturer’s instructions where NADH was subtracted from NAD total to calculate NAD^+^. The ratio of NAD^+^/NADH as well as total NAD and NADH was plotted.

### Mitochondrial respirometry

#### Mitochondrial media

Media were prepared as described in (*109*). Briefly, solution A contained 250 mM sucrose, bovine serum albumin (BSA; 1 g/liter), 0.5 mM Na_2_EDTA, and 10 mM tris-HCl (pH 7.4). Solution B contained 20 mM taurine, 15 mM phosphocreatine, 20 mM imidazole, 0.5 mM dithiothreitol (DTT), 10 mM CaEGTA, 5.77 mM ATP, 6.56 mM MgCl_2_, and 50 mM K-MES (pH 7.1). Solution C contained 0.5 mM EGTA, 60 mM K-lactobionate, 20 mM taurine, 10 mM KH_2_PO₄, 3 mM MgCl_2_, 110 mM sucrose, fatty acid–free BSA (1 g/liter), and 20 mM Hepes (pH 7.1).

#### Tissue permeabilization

Protocol was adjusted from (*109*). Briefly, cortical pieces (approximately 1 mm by 1 mm by 2 mm) were quickly removed and placed in cold solution A [250 mM sucrose, BSA (1 g/liter), 0.5 mM Na_2_EDTA, and 10 mM tris-HCl (pH 7.4)]. Then, cortical tissue was weighed for normalization of oxygen consumption and transferred into 2-ml tubes with 1 ml of cold solution B [20 mM taurine, 15 mM phosphocreatine, 20 mM imidazole, 0.5 mM DTT, 10 mM CaEGTA, 0.1 μM free Ca, 5.77 mM ATP, 6.56 mM MgCl_2_, and 50 mM K-MES (pH 7.1)]. The medium was replaced by 2 ml of cold solution B complemented with 20 μl of a freshly prepared saponin solution (5 mg/ml). After 30 min at 4°C under gentle agitation on an orbital shaker, samples were rinsed in cold solution C [0.5 mM EGTA, 60 mM K-lactobionate, 20 mM taurine, 10 mM KH_2_PO_4_, 3 mM MgCl_2_, 110 mM sucrose, fatty acid–free BSA (1 g/liter), and 20 mM Hepes (pH 7.1)] three times for 2 min each and further incubated on ice until measurements were taken.

#### Oxygen consumption measurement

Oxygen consumption of intact permeabilized cortical sections was measured using a respirometer (Oxygraph-2k, Oroboros Instruments). Measurements were performed under continuous stirring in 2 ml of solution C at 37°C. The solution was equilibrated in air for at least 30 min, and the permeabilized cortical tissue was transferred into the instrument chambers. Mutant mice with respective controls were run in parallel in the instrument’s two chambers simultaneously to minimize day-to-day variability. Only after stabilization of the initial mitochondrial oxygen consumption was mitochondrial respiration stimulated by successive addition of substrates and inhibitors: First, 5 μl of 2 M pyruvate and 5 μl of 800 mM malate were added. Second, 10 μl of 500 mM adenosine 5′-diphosphate (with 300 mM free Mg^2+^) was added to measure O_2_ consumption under a normal phosphorylating state. Third, 5 μl of 4 mM cytochrome c was added to check for mitochondrial membrane integrity; any samples that responded with a significant increase in O_2_ consumption were removed from further analysis. Fourth, 10 μl of 2 M glutamate was added. Fifth, 20 μl of 1 M succinate was added. Sixth, 1 μl of 5 mM oligomycin, an ATP synthase inhibitor, was added. Then, maximum mitochondrial capacity was assessed by adding gradual volumes of 1 mM carbonyl cyanide *p*-trifluoromethoxyphenylhydrazone (FCCP) until maximum oxygen consumption was reached. Then, 1 μl of 1 mM rotenone, a complex one inhibitor, was added to measure complex two activity. Last, 1 μl of 5 mM antimycin A, a complex three inhibitor, was added to measure nonmitochondrial O_2_ consumption (residual oxygen flux) due to cytosolic oxidases. Residual oxygen flux was subtracted from all other measurements to report baseline mitochondrial oxygen consumption. Mitochondrial oxygen consumption was calculated using DataGraph software from the manufacturer (Oroboros Instruments). Spare mitochondrial capacity in the brain tissue was measured after titration of a protonophore (FCCP) and subtracting the baseline mitochondrial oxygen consumption.

#### Maximal ETS measurement

Maximal electron transport system (ETS) activity was measured in permeabilized cortical tissue (~5 mg) with saturating concentrations of NADH (10 mM). The tissue was permeabilized by homogenizing the tissue in mitochondrial isolation buffer [310 mM sucrose, 20 mM tris, and 1 mM EGTA (pH 7.2)] with a 23-gauge syringe multiple times, followed by two freeze-thaw cycles. The experiment was performed after calibrating the same respirometer (Oxygraph-2k, Oroboros Instruments) used previously, followed by adding 2 μl of 8.1 mM digitonin (Sigma-Aldrich, D 5628). Then, 10 μl of 4 mM cytochrome c (Sigma-Aldrich, C 7752) and 20 μl of 10 mM freshly prepared NADH (Sigma-Aldrich, 43420) were added until saturating concentrations (saturating concentrations for cortical tissue were identified in preliminary experiments). After the chamber reached equilibrium, saturating levels of succinate were added by injecting 20 μl of 1 M succinate to drive maximal ETS function. Last, 1 μl of 5 mM antimycin A was added to measure nonmitochondrial O_2_ consumption (residual oxygen flux). Residual oxygen flux was subtracted from all other measurements to report mitochondrial oxygen consumption. Mitochondrial oxygen consumption relative to the wet mass of the tissue was calculated using DataGraph software from the manufacturer (Oroboros Instruments).

### Statistical analyses

Mean life span was assessed, and survivorship was analyzed using log-rank test or Cox proportional hazard analysis where denoted. Two-group comparisons were made using two-tailed, unpaired Student’s *t* test unless otherwise stated. For comparisons of multiple factors (for example, phase*genotype or sex × genotype), two-way ANOVA was reported, followed by Sidak’s posttest if interaction between main effects was significant.

Numbers of mice were estimated to be sufficient to detect statistically meaningful differences of at least 20% between or among groups using standard power calculations with α = 0.05 and a power of 0.8 on the basis of similar experiments conducted in our group. Homogeneity of variance and normality of residuals were assessed, and appropriate corrections were made if necessary. All experiments were performed in a randomized and blinded fashion when possible. Data were analyzed statistically using GraphPad Prism 9.4, and outliers were removed from analysis based on a Grubb’s test. The value of α was 0.05, and data were expressed as *P < 0.05, **P < 0.01, ***P < 0.001, and ****P < 0.0001. The number of animals is reported at the bottom of the bars for each condition or in the figure legends. All error bars correspond to SD except for longitudinal glucose and insulin sensitivity where SEM is reported. ANCOVA analyses were plotted with 95% confidence interval bands. Detailed *P* values for nonsignificant comparisons, test statistic values, and degrees of freedom are included in table S1.

## Notes

View/request a protocol for this paper from *Bio-protocol*.
