# Supplementary material for: Reduced insulin signaling in neurons induces sex-specific health benefits
Source: Sci Adv. 2023 Feb 22;9(8):eade8137. doi: 10.1126/sciadv.ade8137 (PMC9946356; doi:10.1126/sciadv.ade8137)
Supplement: Supplementary file 1 — Figs. S1 to S20 Legends for tables S1 to S4 [file sciadv.ade8137_sm.pdf]

Science Advances
AAAS

Supplementary Materials for

Reduced insulin signaling in neurons induces sex-specific health benefits

Maarouf Baghdadi *et al.*

Corresponding author: Linda Partridge, linda.partridge@age.mpg.de

Sci. Adv. **9**, eade8137 (2023)
DOI: 10.1126/sciadv.ade8137

The PDF file includes:

Figs. S1 to S20
Legends for tables S1 to S4

Other Supplementary Material for this manuscript includes the following:

Tables S1 to S4

Supplementary Figure 1

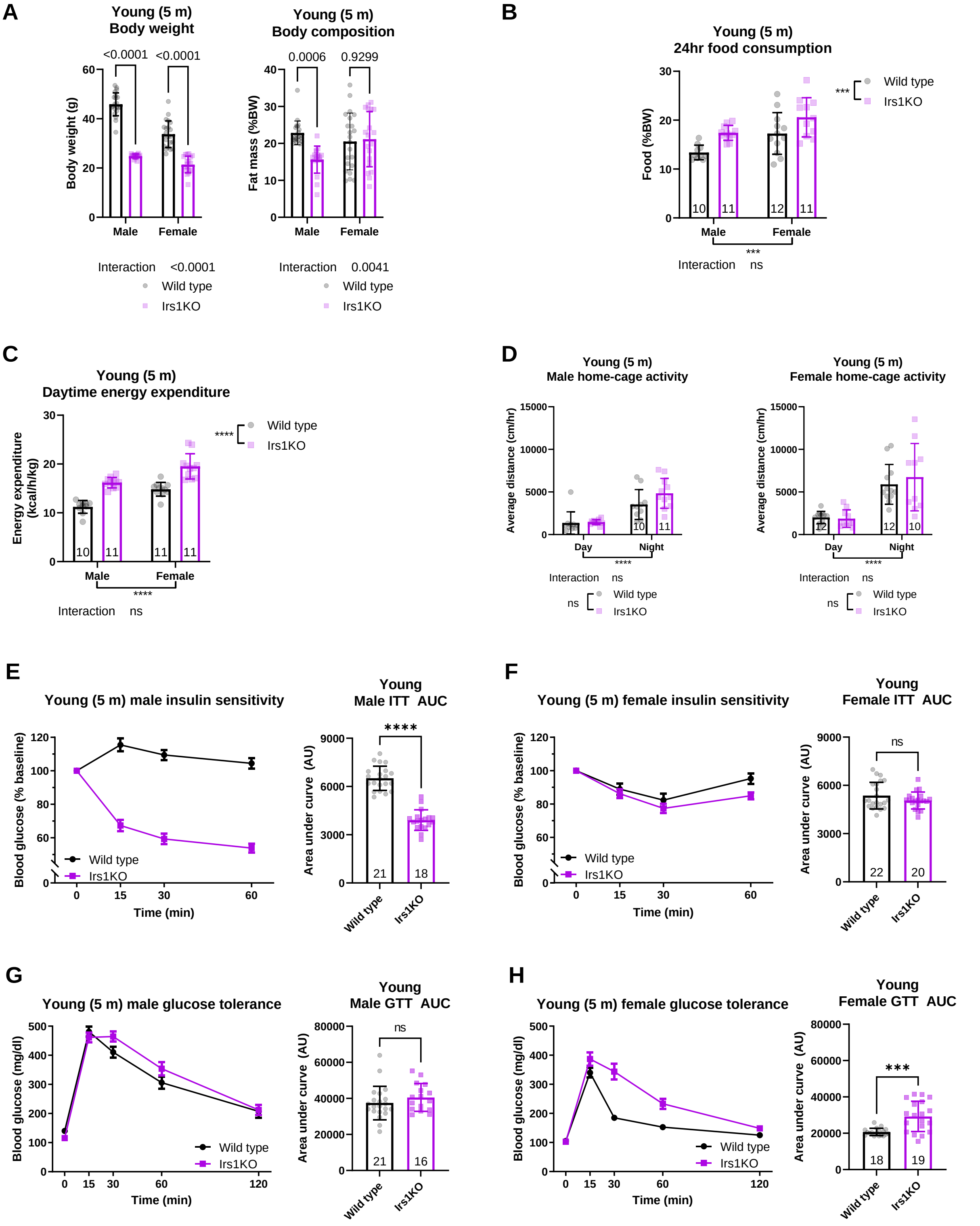

Supplementary Figure 1: Characterisation of young Irs1KO

(A) Body weight and body composition of Irs1KO mice measured at young age (5 months) (Wild type males n=21, Irs1KO males n=18, Wild type females n=22, Irs1KO females n=19). **(B)** Measurement of food consumption of young Irs1KO and wild type

mice revealed a significant increase in food consumption of single housed Irs1KO male and female mice. **(C)** Young body weight normalised energy expenditure of singly housed male and female mice during daytime as assessed by metabolic chambers showing an increase in energy expenditure in Irs1KO mice. **(D)** Spontaneous activity of Irs1KO single housed mice during their inactive cycle or daytime showed no significant difference in activity at young age (Wild type males n=10, Irs1KO males n=11, Wild type females n=12, Irs1KO females n=10). Insulin tolerance test (ITT) of young male **(E)** Irs1KO mice revealed a clear enhanced systemic insulin sensitivity in male Irs1KO mice, while no difference was observed between female **(F)** Irs1KO mice and their wild type littermates. **(G)** Glucose tolerance test (GTT) did not show any significant difference in glucose tolerance in young male Irs1KO and wild type littermate mice. **(H)** AUC analysis of GTT in young female Irs1KO mice revealed a significantly reduced glucose tolerance in Irs1KO females compared to wild type littermate mice. All error bars correspond to standard deviation except for longitudinal glucose and insulin sensitivity where standard error of the mean is reported. Number of animals reported at the bottom of the bars for each condition. Detailed statistical values found in Table S1.

Supplementary Figure 2

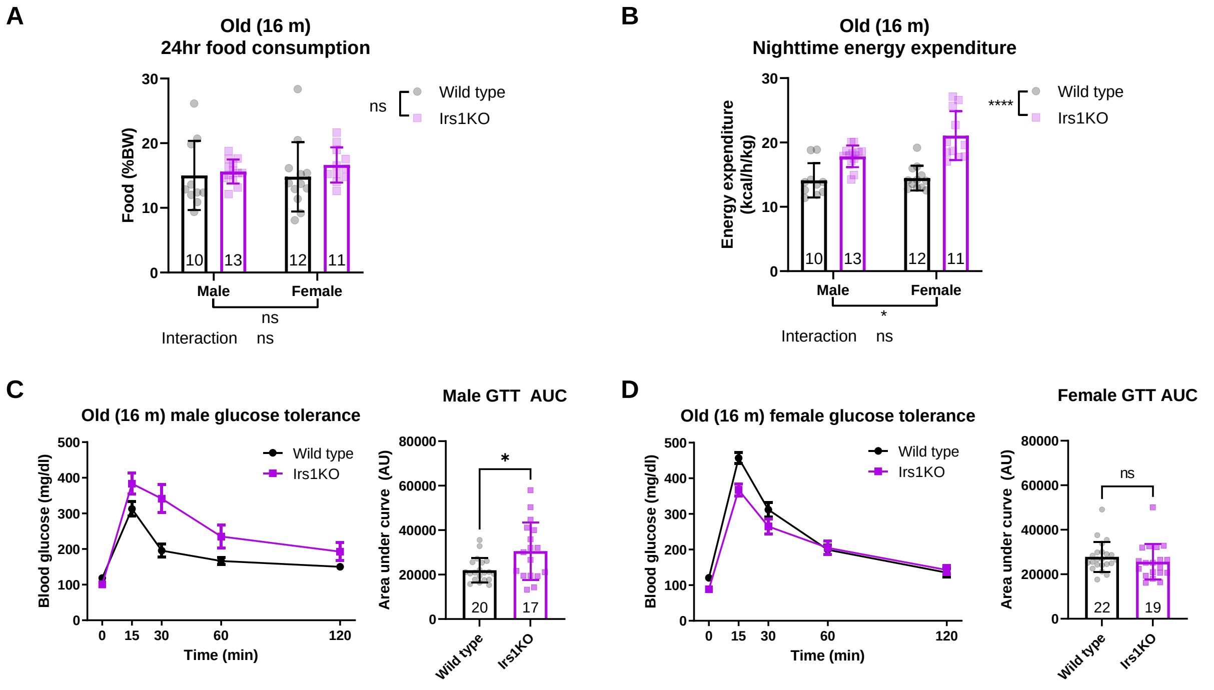

Supplementary Figure 2: Additional parameters of old Irs1KO

(A) Old (16 months) Irs1KO and wild type food consumption measured in single housed animals shows no significant differences. **(B)** Body weight normalised energy expenditure of old Irs1KO mice during nighttime in individually housed animals showing significant increase in old Irs1KO mice. Glucose tolerance test (GTT) was administered to old male **(C)** and female **(D)** Irs1KO mice, by measuring blood glucose levels in response to a body weight adjusted glucose bolus. Old male Irs1KO mice showed significant reduction in glucose sensitivity. Detailed statistical values found in Table S1.

Supplementary Figure 3

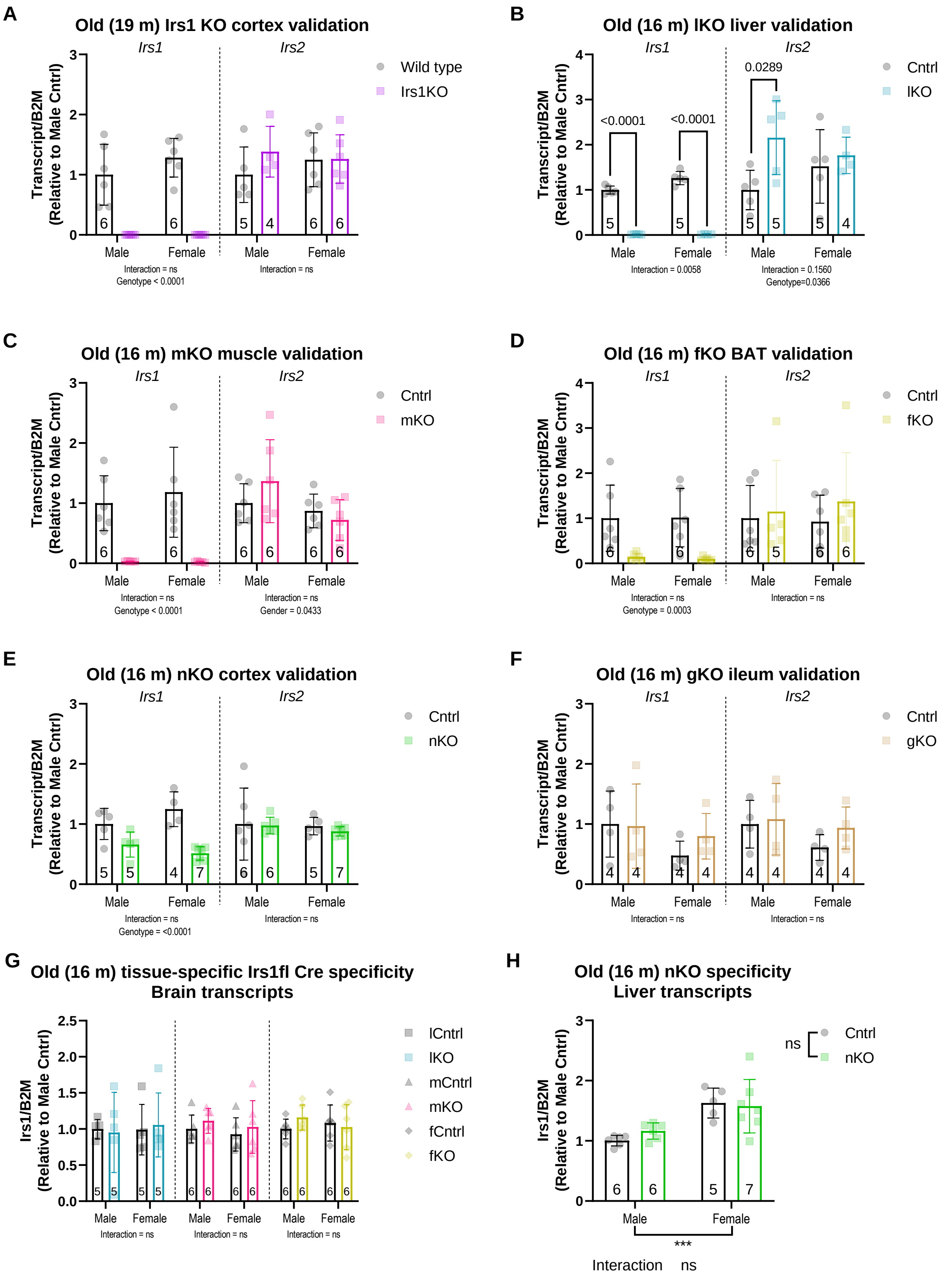

Supplementary Figure 3: Validation of mouse models used in the study

(A) Quantitative real-time PCR of cortical tissue in old (19 months) Irs1KO mice shows depletion of Irs1 transcripts. Irs2 transcript levels show no compensatory effect. **(B)** Liver samples of lKO mice show depletion of Irs1 transcripts and

compensatory Irs2 upregulation in male lKO mice. **(C)** Hindlimb muscle samples revealed depletion of Irs1 transcript levels in mKO mice, but no effect on Irs2 transcript levels. **(D)** Supraclavicular brown adipose tissue (BAT) samples of fKO mice show depletion of Irs1 transcripts with no compensatory Irs2 upregulation. **(E)** Cortex samples of nKO mice show significant reduction but not depletion of Irs1 transcripts in nKO mice, with no effect on Irs2 transcript levels. **(F)** lKO, mKO and fKO cortical samples used to assess Irs1 transcript levels did not reveal any non-specific Irs1 deletion in brain tissue. **(G)** Liver samples from nKO mice not showing any non-specific Irs1 deletion in liver tissue. All error bars correspond to standard deviation. Number of animals reported at the bottom of the bars or in figure legends. Detailed statistical values found in Table S1.

Supplementary Figure 4

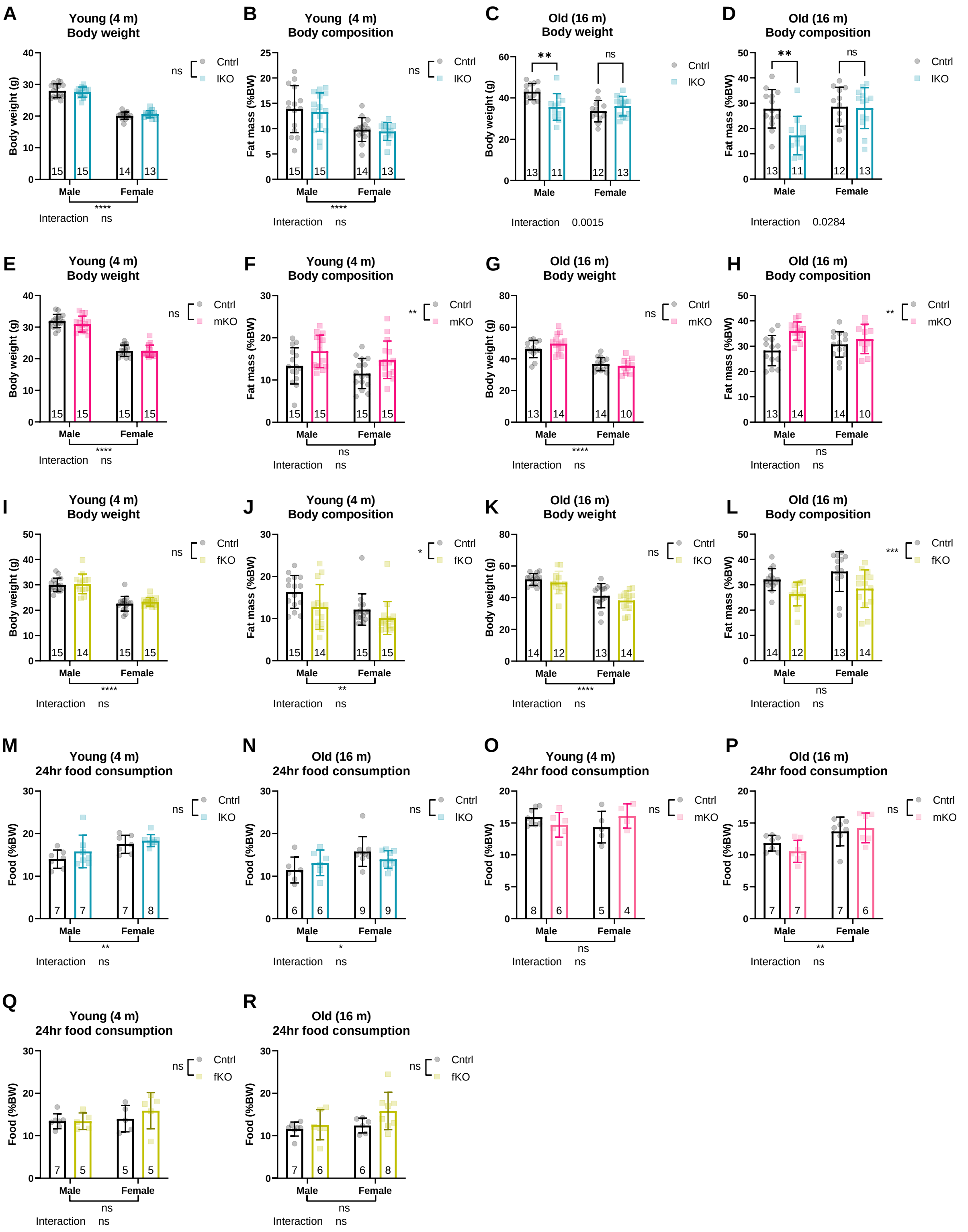

Supplementary Figure 4: Body weight and composition of tissue-specific Irs1KO mice

Body weight **(A)** and body composition **(B)** of young (4 months) male and female lKO mice revealed no significant difference between lKO and control littermates. **(C)** Body weight of old (16 months) male and female lKO revealed a sex-specific significant

reduction in body weight of male lKO mice. **(D)** Body composition at old age also revealed sex-specific significant reduction in body weight adjusted fat mass of male lKO mice. Body weight of young **(E)** (4 months) and old **(F)** (16 months) mKO mice showed no significant difference. Body composition in young **(G)** and old **(H)** mKO mice revealed a significant age-independent increase in fat mass of mKO mice compared to control littermates. Body weight of young **(I)** (4 months) and old **(K)** (16 months) fKO mice showed no significant difference. Body composition in young **(J)** and old **(L)** fKO mice revealed a significant age-independent decrease in fat mass of fKO mice compared to control littermates. Measurement of food consumption of young lKO **(M)** and old lKO **(N)** revealed no significant difference in food consumption of single housed animals relative to the corresponding littermate controls. Measurement of food consumption of young mKO **(O)** and old mKO **(P)** revealed no significant difference in food consumption of single housed animals relative to the corresponding littermate controls. Measurement of food consumption of young fKO **(Q)** and old fKO **(R)** revealed no significant difference in food consumption of single housed animals relative to the corresponding littermate controls. Number of animals reported at the bottom of the bars for each condition. Detailed statistical values found in Table S1.

Supplementary Figure 5

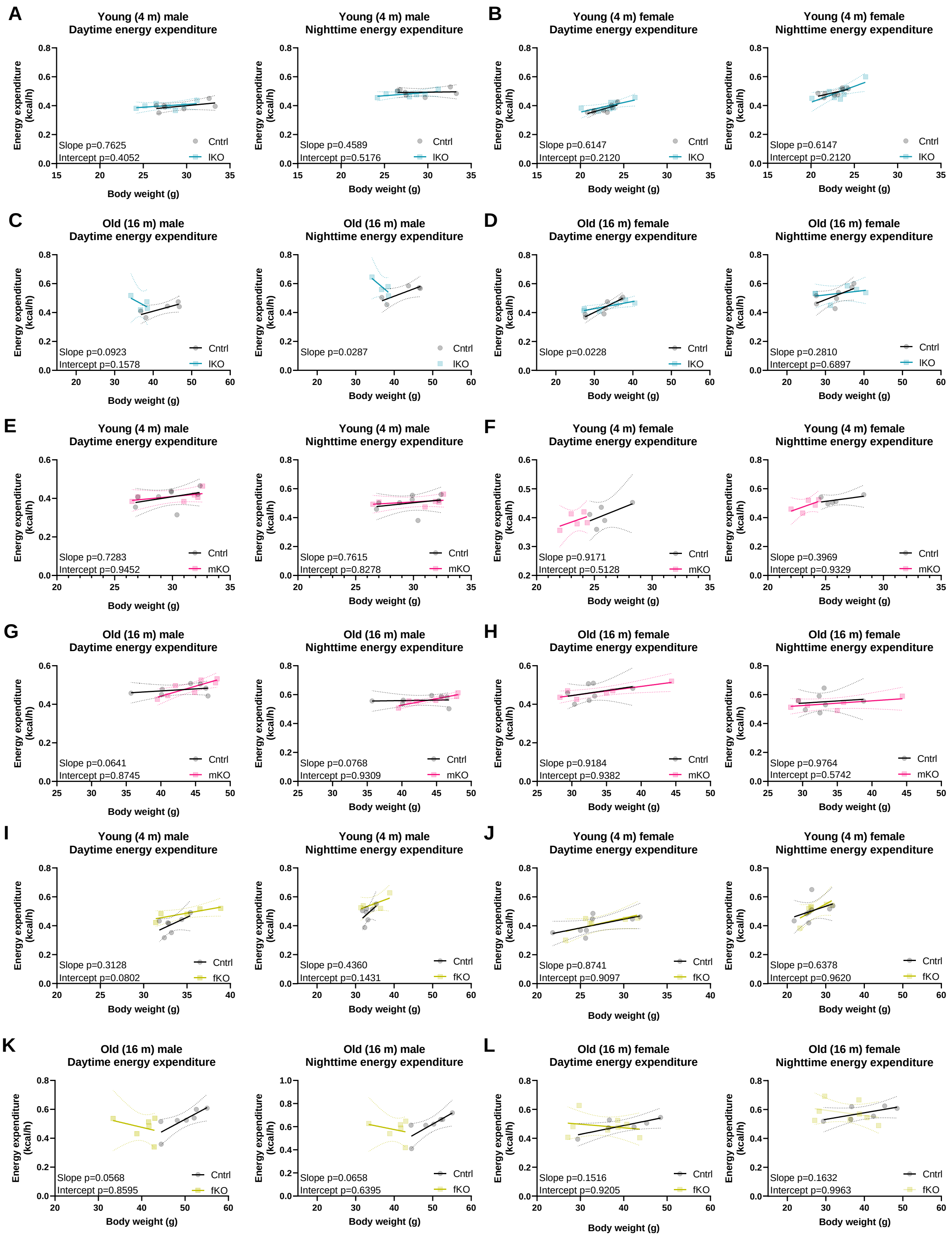

Supplementary Figure 5: Energy expenditure of tissue-specific Irs1KO mice

Daytime and nighttime energy expenditure were analysed by linear regression of energy expenditure by body weight (ANCOVA). No difference in energy expenditure was detected in young male **(A)** or female **(B)** IKOs (male control and IKO n=7,

female controls and lKO n=8) or old male **(C)** and female **(D)** lKOs (male control n=5 and lKO n=4, female controls n=7 and lKO n=6). No difference in energy expenditure was detected in young male **(E)** and female **(F)** mKOs (male control n=8 and mKO n=6, female controls and mKO n=5) or old male **(G)** and female **(H)** mKOs (male control and mKO n=7, female control n=7 and mKO n=6). No difference in energy expenditure was detected in young male **(I)** and female **(J)** fKOs (male control n=7 and fKO n=5, female control n=8 and fKO n=6) or old male **(K)** and female **(L)** fKOs (male control n=7 and fKO n=6, female control n=6 and fKO n=8). Detailed statistical values found in Table S1.

Supplementary Figure 6

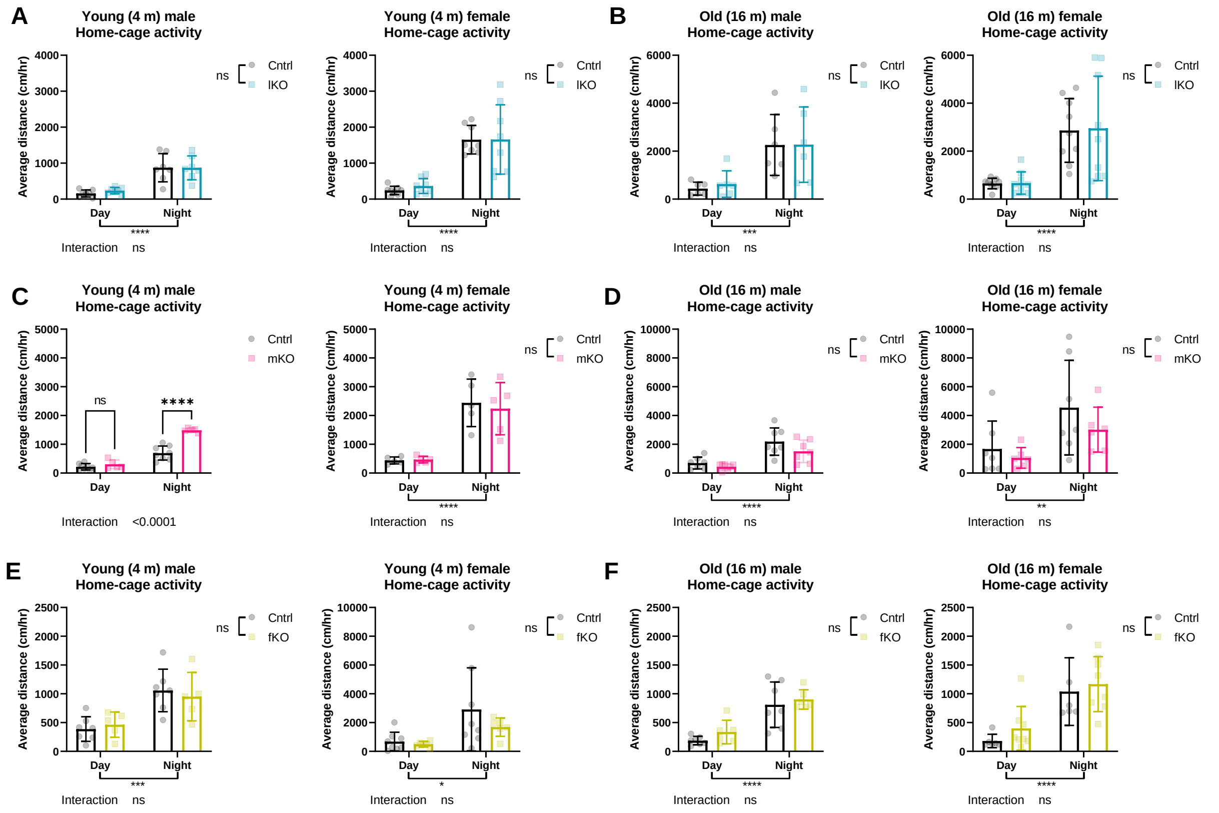

Supplementary Figure 6: Locomotor activity of tissue-specific Irs1KO mice

Measurement of spontaneous locomotor activity of singly housed young lKO **(A)** (male control and lKO n=7, female controls and lKO n=8) and old lKO **(B)** (male control and lKO n=6, female controls and lKO n=9) mice during daytime and nighttime did not reveal any significant difference due to genotype. Spontaneous locomotor activity of singly housed young male mKO mice **(C)** (control n=8 and mKO n=5) revealed higher activity levels during nighttime compared to controls. Spontaneous activity of young female mKO **(C)** (control and mKO n=5), old male mKO **(D)** (control and mKO n=7) or old female mKO **(D)** (control n=7 and mKO n=6) mice during daytime and nighttime did not reveal any significant difference due to genotype. Spontaneous locomotor activity of singly housed young fKO **(E)** (male control n=7 and fKO n=5, female controls n=8 and fKO n=6) and old fKO **(F)** (male control n=7 and fKO n=6, female controls n=6 and fKO n=8) mice during daytime and nighttime did not reveal any significant differences. Detailed statistical values found in Table S1.

Supplementary Figure 7

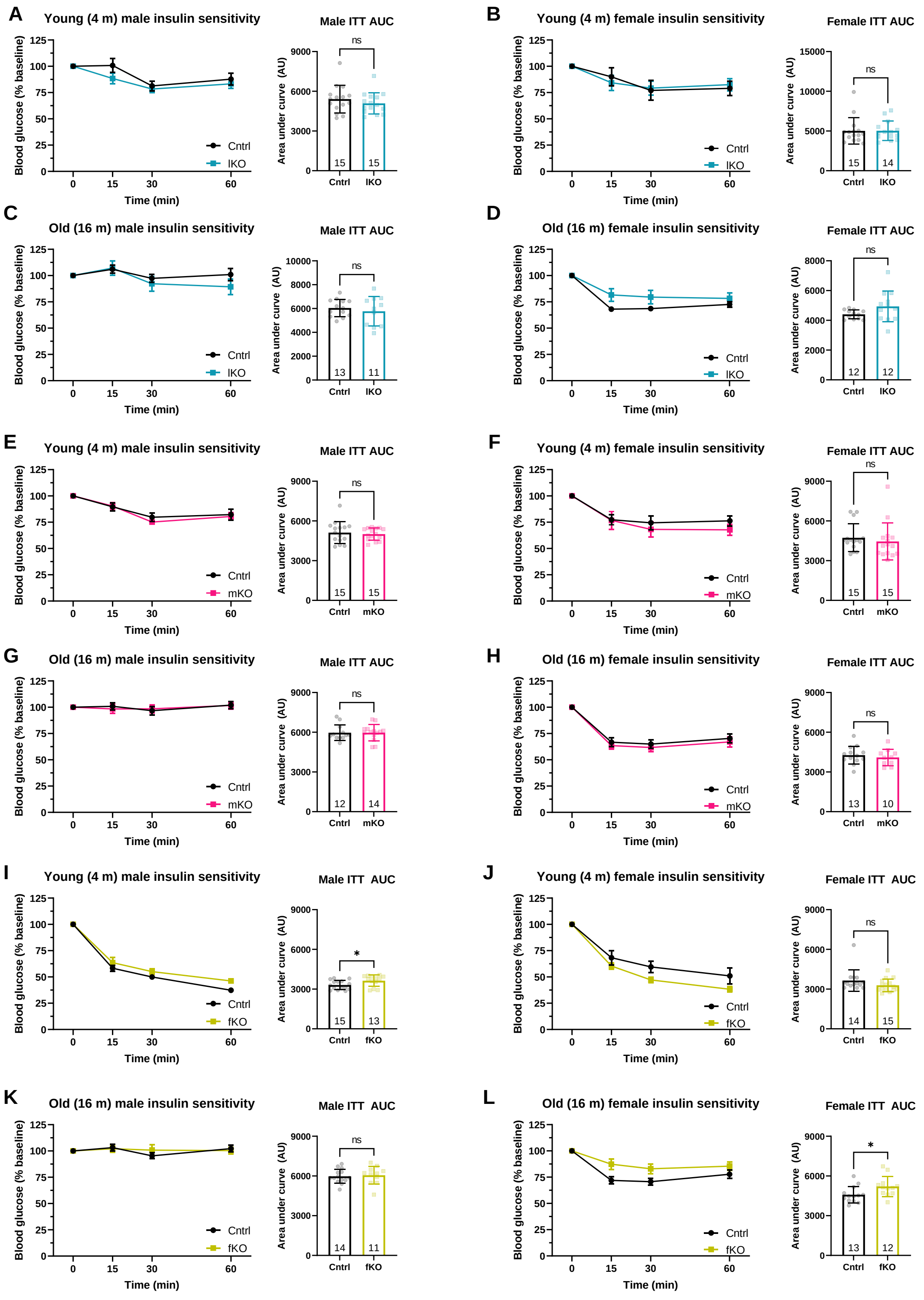

Supplementary Figure 7: Insulin tolerance test of tissue-specific Irs1KO mice

Insulin tolerance test (ITT) revealed no significant difference in insulin sensitivity of young male lKO **(A)**, young female lKO **(B),** old male lKO **(C)** and old female lKO **(D)** compared to their respective control littermates as assessed by AUC analysis. No

significant difference in insulin sensitivity of young male mKO **(E)**, young female mKO **(F),** old male mKO **(G)** and old female mKO **(H)** compared to their respective control littermates as assessed by AUC analysis. ITT of young male fKO **(I)** revealed a significant reduction in insulin sensitivity between fKO and control littermates. ITT analysis of young female fKO **(J)** and old male fKO **(K)** did not detect any significant differences between fKO mice and their control littermates. ITT of old female fKO **(L)** revealed a significant reduction in insulin sensitivity between fKO and control littermates. Detailed statistical values found in Table S1.

Supplementary Figure 8

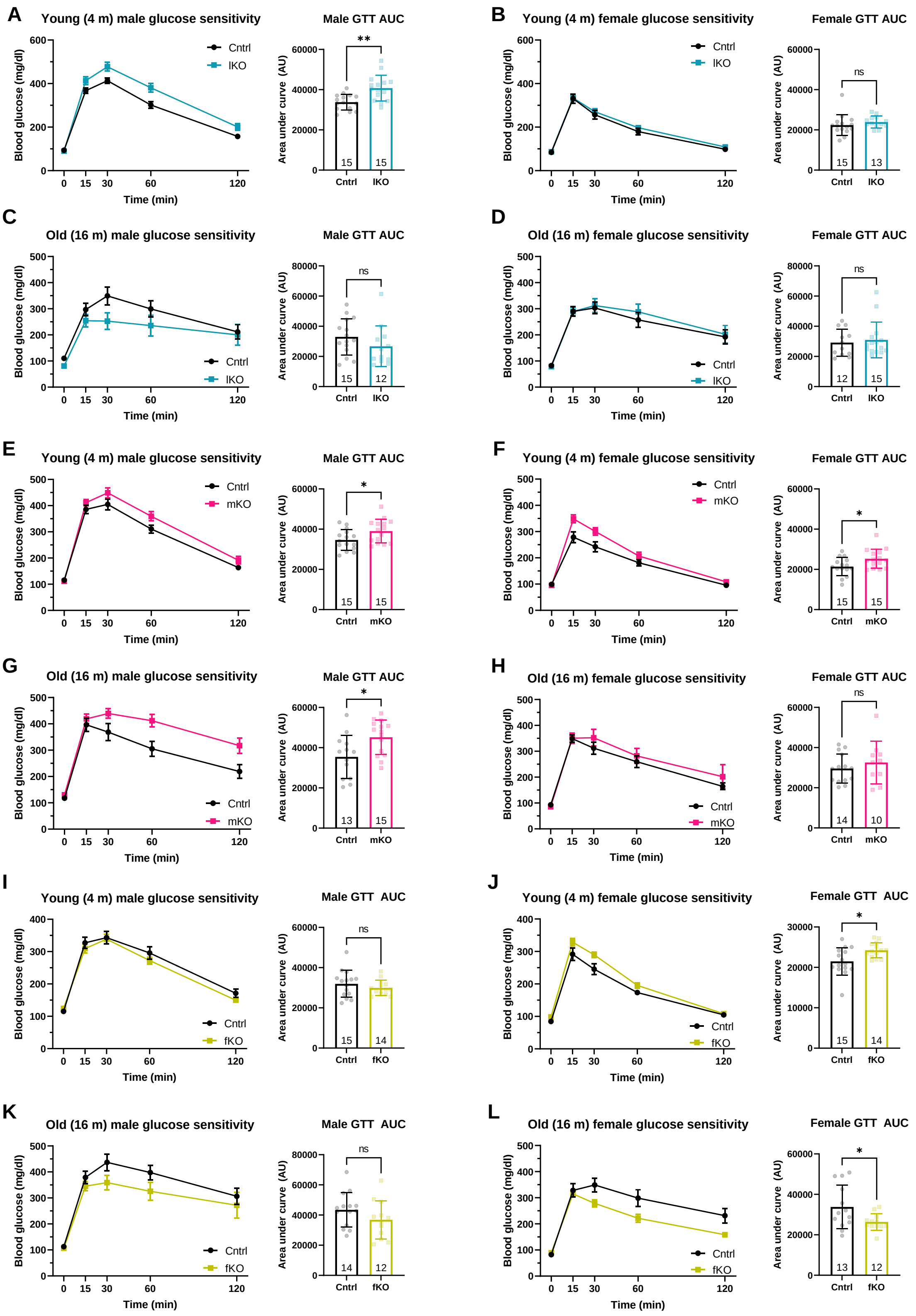

Supplementary Figure 8: Glucose tolerance test of tissue-specific Irs1KO mice (below)

Glucose tolerance test (GTT) revealed a significant reduction in glucose tolerance of young male lKO **(A)** compared to control mice as assessed by AUC analysis. AUC analysis of GTT of young female lKO **(B)**, old male lKO **(C)** and old female lKO **(D)** did not detect any significant differences between lKO mice and their control littermates. GTT revealed a significant reduction in glucose tolerance of young male mKO **(E)**, young female mKO **(F)** and old male mKO **(G)** as assessed by AUC analysis. GTT of old female mKO **(H)** did not detect any significant differences between mKO mice and their control littermates. GTT of young male fKO **(I)** did not detect any significant differences between fKO mice and their control littermates. GTT of young female fKO **(J)** revealed a significant reduction in glucose tolerance as assessed by AUC analysis. GTT of old male fKO **(K)** and old female fKO **(L)** did not detect any significant differences between fKO mice and their control littermates. Detailed statistical values found in Table S1.

Supplementary Figure 9

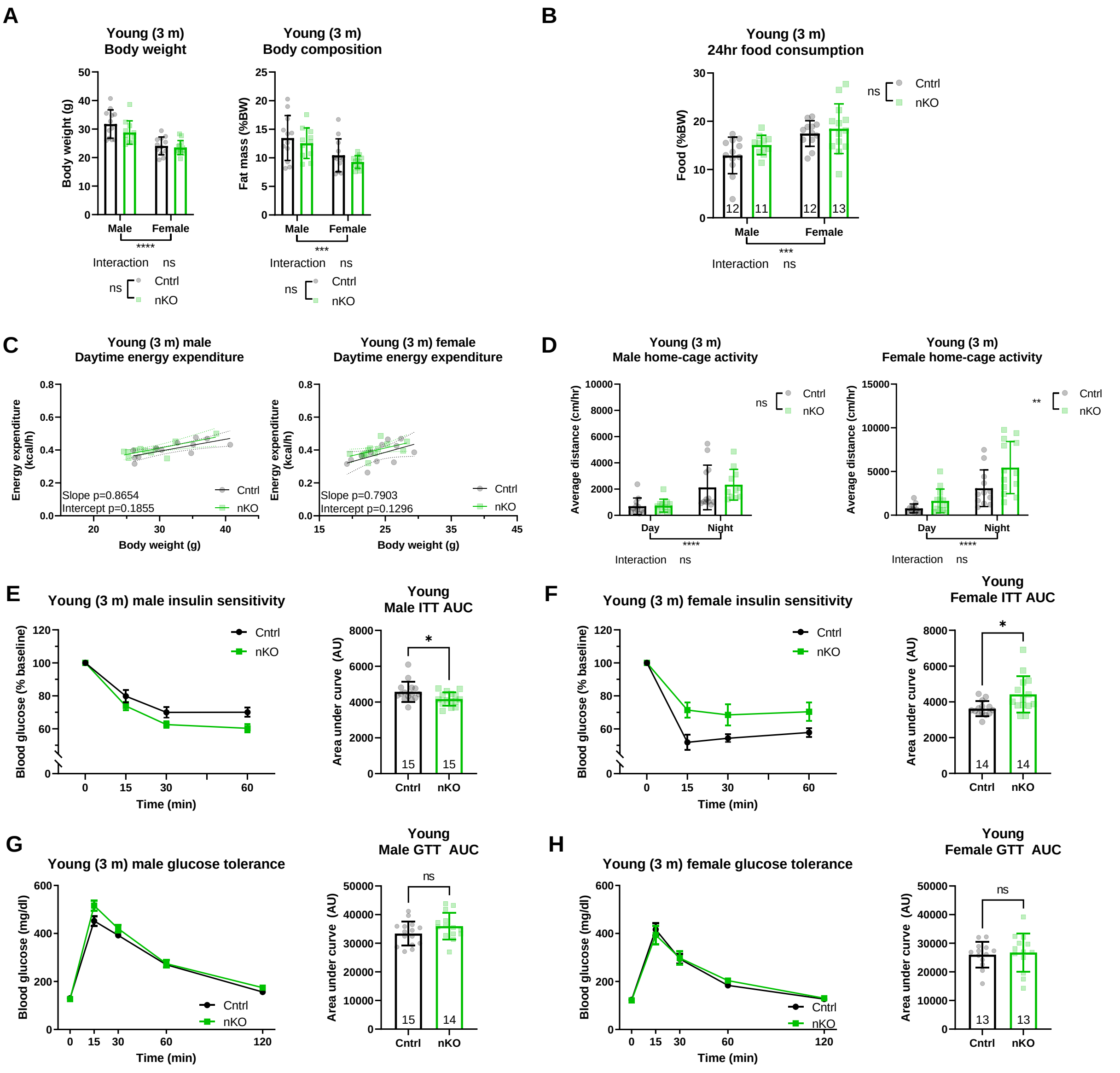

Supplementary Figure 9: Characterisation of young nKO

(A) No significant difference was observed in body weight or body composition at young age (3 months) of nKO and control littermates (male nKO and controls n=15, female nKO and control n=14). **(B)** Measurement of food consumption of young nKO and control mice revealed no significant difference in food consumption of single housed animals. **(C)** Daytime energy expenditure of male nKO and female nKO and control mice was analysed by linear regression of energy expenditure by body weight (ANCOVA). No difference in energy expenditure between young male nKO (n=11) and controls (n=12) or female nKO (n=13) and controls (n=12) was observed. **(D)** No significant difference was observed in spontaneous activity of young single-housed male nKO (n=12) and littermate control (n=11) mice. A significant increase in daytime and nighttime activity was observed between female nKO (n=13) and littermate control (n=12) mice. Insulin sensitivity of male nKO and control mice **(E)** showed a significantly improved insulin sensitivity in male nKO mice. However, a significant reduction in insulin sensitivity was detected between female **(F)** nKO and control mice. Analysis of GTT of male **(G)** and female **(H)** nKO mice did not reveal any significant difference in young nKO glucose tolerance compared to control littermates. All error bars correspond to standard deviation except for longitudinal glucose and insulin sensitivity where standard error of the mean is reported. Number of animals reported at the bottom of the bars for each condition. Detailed statistical values found in Table S1.

Supplementary Figure 10

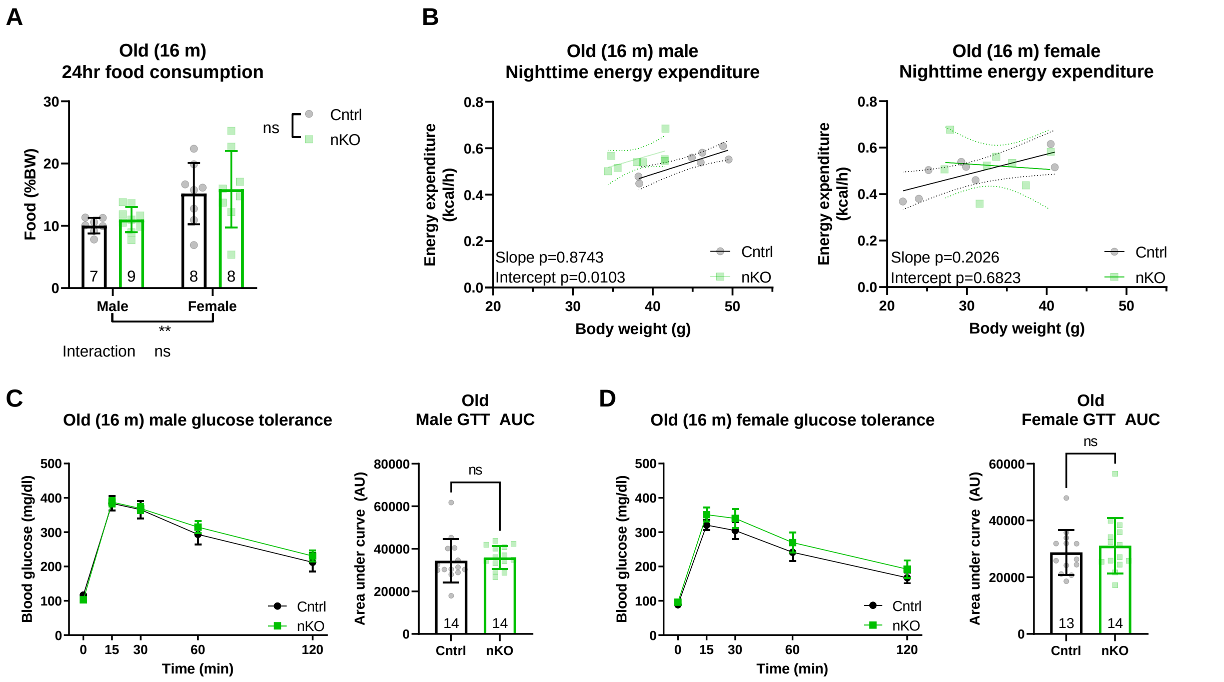

Supplementary Figure 10: Additional parameters of old nKO

(A) No difference in food consumption was detected in single housed old (16 months) nKO and control littermates. **(B)** Nighttime energy expenditure of male and female mice was analysed by linear regression of energy expenditure by body weight (ANCOVA). Unlike female nKO mice (n=8 female control and nKO mice) where no significant difference between intercepts was detected, male nKO (n=8) mice showed significant increase in energy expenditure compared to male controls (n=7). Analysis of GTT did not detect any significant difference between the ability of old male **(C)** and female **(D)** nKO mice compared to their respective littermate controls in lowering blood glucose levels. All error bars correspond to standard deviation except for longitudinal glucose sensitivity where standard error of the mean is reported. Number of animals reported at the bottom of the bars for each condition. Detailed statistical values found in Table S1.

Supplementary Figure 11

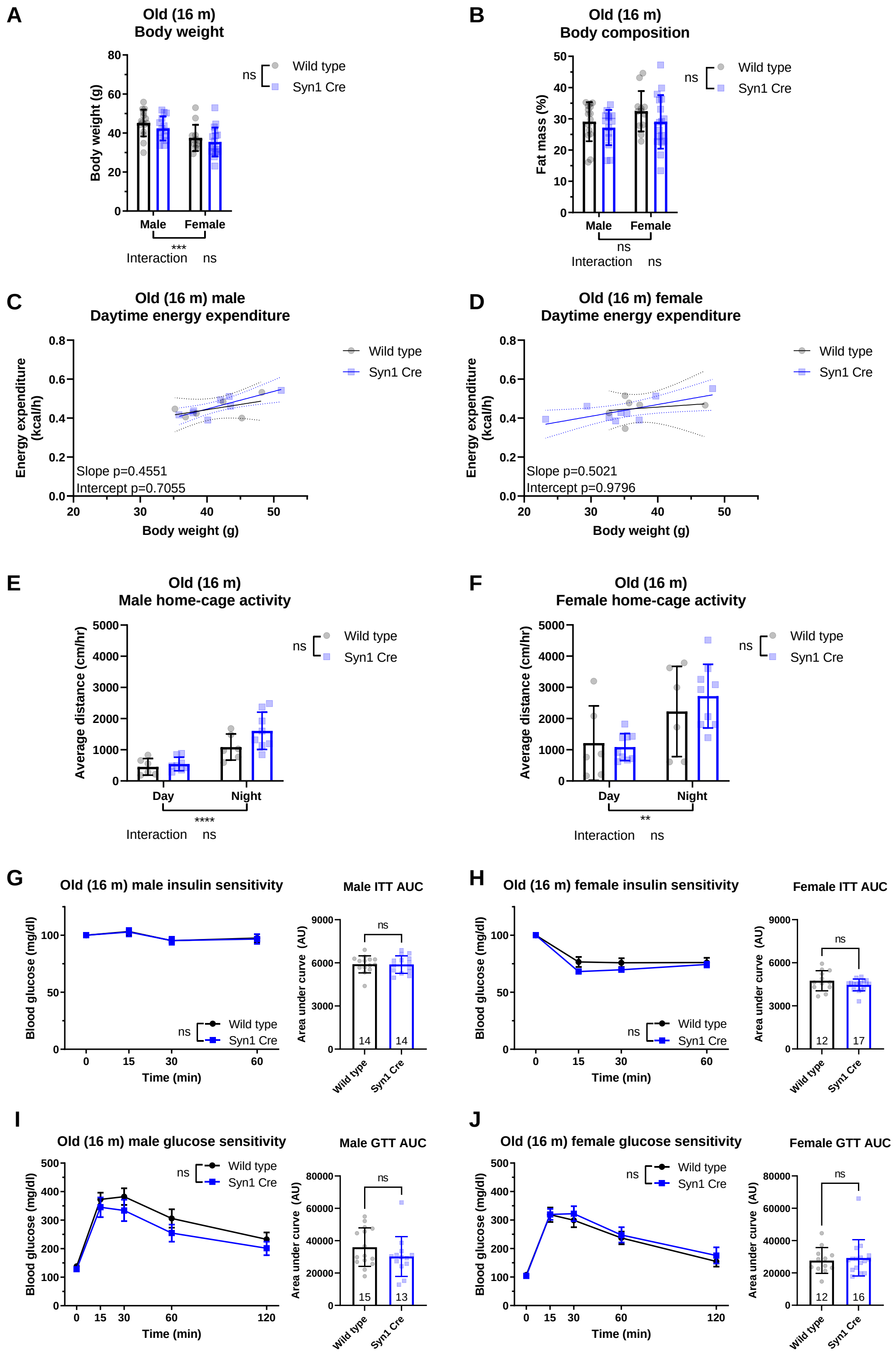

Supplementary Figure 11: Neuronal Syn1Cre expression does not affect peripheral metabolism (below)

(A) No significant difference was observed in body weight or **(B)** body composition at old age (16 months) of Syn1Cre and wild type littermates (male wild type n=15 and Syn1Cre n=14, female wild type n=12 and Syn1Cre n=17). Daytime energy expenditure of **(C)** male and **(D)** female Syn1Cre and wild type mice was analysed by linear regression of energy expenditure by body weight (ANCOVA). No difference in energy expenditure between old male Syn1Cre (n=8) and wild type (n=6) as well as between female Syn1Cre (n=9) and wild type (n=6) was observed. No significant difference was observed in spontaneous activity of old single-housed **(E)** male Syn1Cre (n=8) and littermate wild type (n=6) mice or female **(F)** Syn1Cre (n=9) and wild type (n=6) mice. Insulin sensitivity of old **(G)** male or **(H)** female Syn1Cre and wild type mice showed no significant difference in insulin sensitivity. Analysis of GTT of male **(I)** and female **(J)** Syn1Cre mice did not reveal any significant difference in old Syn1Cre glucose tolerance compared to wild type littermates. All error bars correspond to standard deviation except for longitudinal glucose and insulin sensitivity where standard error of the mean is reported. Number of animals reported at the bottom of the bars for each condition. Detailed statistical values found in Table S1.

Supplementary Figure 12

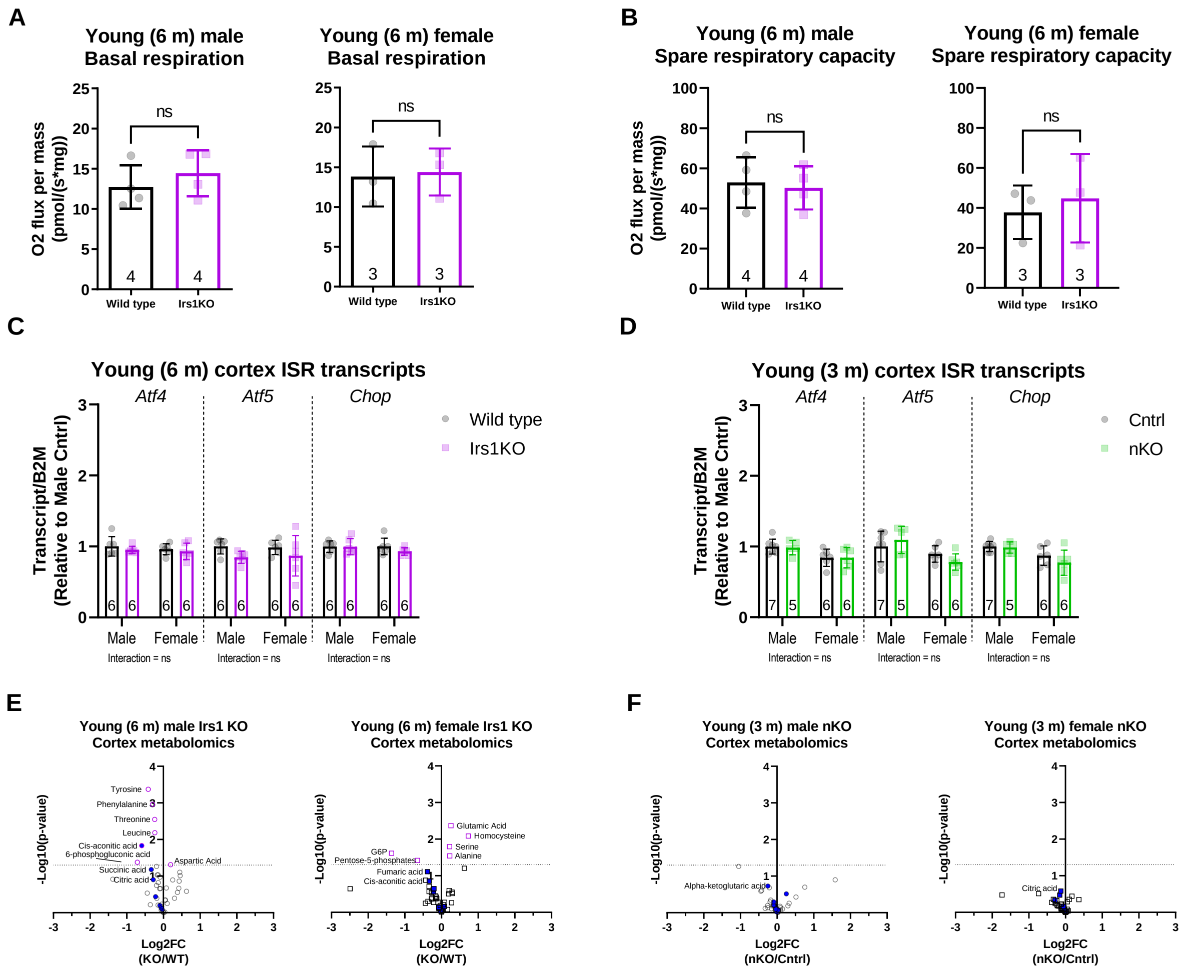

Supplementary Figure 12: No activation of ISR in brains of young Irs1KO and nKO mice

(A) Basal oxygen consumption of brain tissue showed no difference in the basal respiration of mitochondria of young (6 months) male or female Irs1KO mice. **(B)** Mitochondrial spare respiratory capacity in brain tissue revealed no significant difference in young male or female Irs1KO mitochondrial function. **(C)** Quantitative real-time PCR was performed on brains of young male and female Irs1KO mice and their wild type littermates to measure transcript levels of several integrated stress response (ISR) markers revealed no significant differences. **(D)** Transcripts of ISR markers were measured in brains of young (3 months) male and female nKO mice and their control littermates showed no significant differences. **(E)** Semi-targeted metabolomics revealed down-regulation in metabolites in male Irs1KO mice and up-regulation in metabolites in female Irs1KO mice (male wild type and Irs1KO n=6, female wild type and Irs1KO n=6). Steady-state TCA cycle intermediates were labelled in blue, highest significance were highlighted. **(F)** No significant change in metabolites in either male or female young nKO brain tissue (male control n=7 and nKO n=5, female control and nKO n=6). Steady-state TCA cycle intermediates were labelled in blue, highest significance were highlighted. All error bars correspond to standard deviation. Detailed statistical values found in Table S1. Full metabolites measured in Table S2.

Supplementary Figure 13

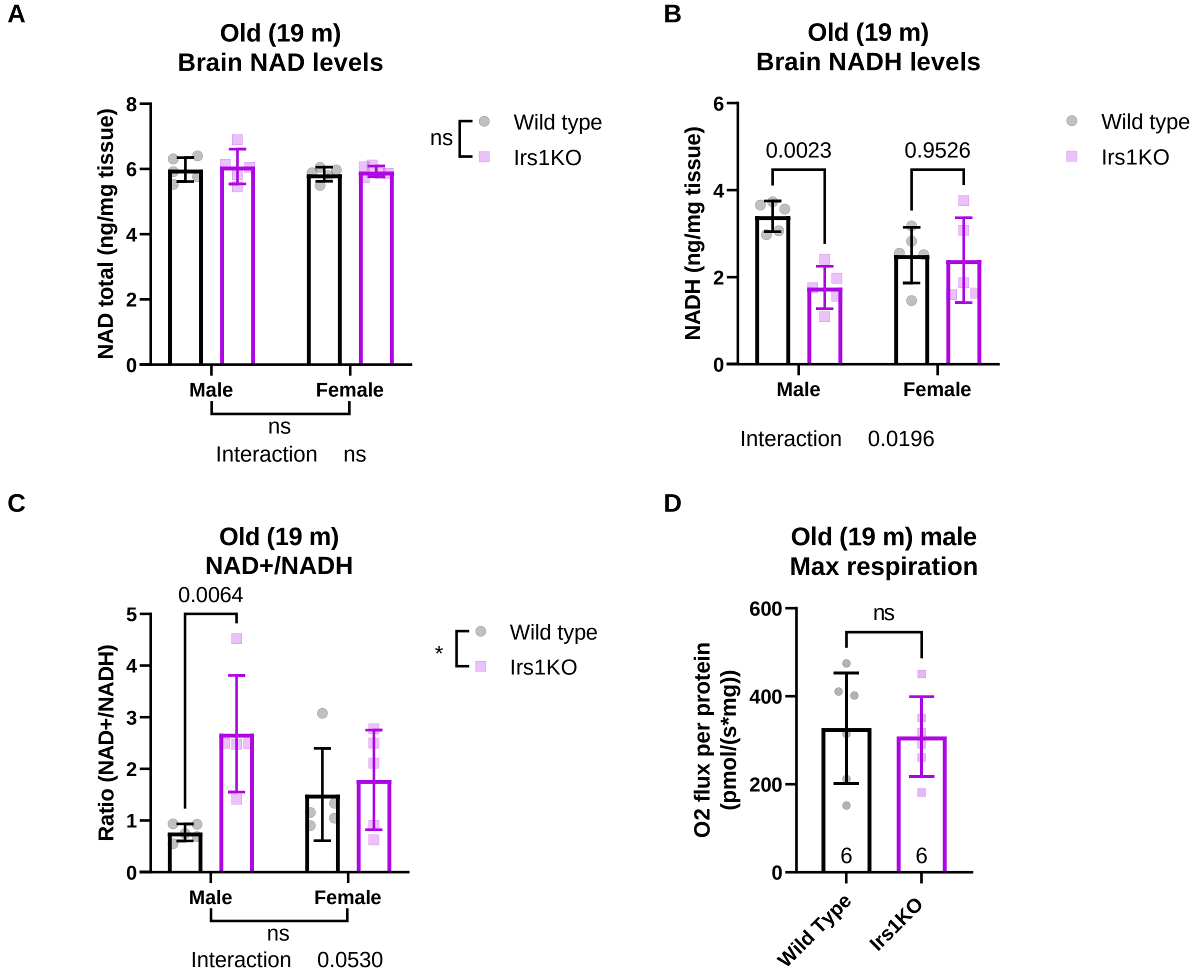

Supplementary Figure 13: IRS1 deletion leads to reduced NADH levels in the brain of old male Irs1KO mice (below)

(A) Total NAD levels were measured in brain cortex samples of old (19 months) Irs1KO mice did not detect a significant difference. **(B)** Brain NADH levels revealed a sex-specific significant reduction in male Irs1KO mice. **(C)** The ratios of NAD+/NADH were plotted for old Irs1KO mice that showed a higher ratio in male Irs1KO mice. **(D)** Maximal oxygen consumption was assessed using a respirometer after saturating cytochrome c, succinate and NADH. All error bars correspond to standard deviation. Detailed statistical values found in Table S1.

Supplementary Figure 14

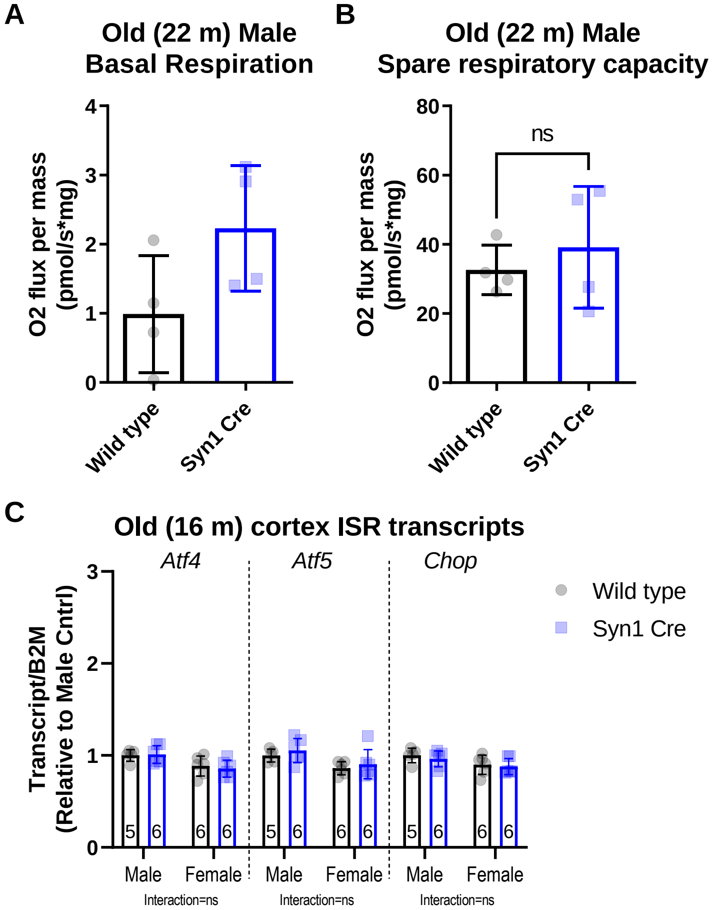

Supplementary Figure 14: Syn1Cre expression does not lead to brain ISR activation

(A) Basal oxygen consumption of brain tissue showed no difference in the basal respiration of mitochondria of old (22 months) male Syn1Cre mice compared to their wild type littermates. **(B)** Mitochondrial spare respiratory capacity in brain tissue revealed no significant difference in old male Syn1Cre mitochondrial function. **(C)** Quantitative real-time PCR was performed on brains of old (16 months) male and female Syn1Cre mice and their wild type littermates to measure transcript levels of several integrated stress response (ISR) markers revealed no significant differences. All error bars correspond to standard deviation. Detailed statistical values found in Table S1.

Supplementary Figure 15

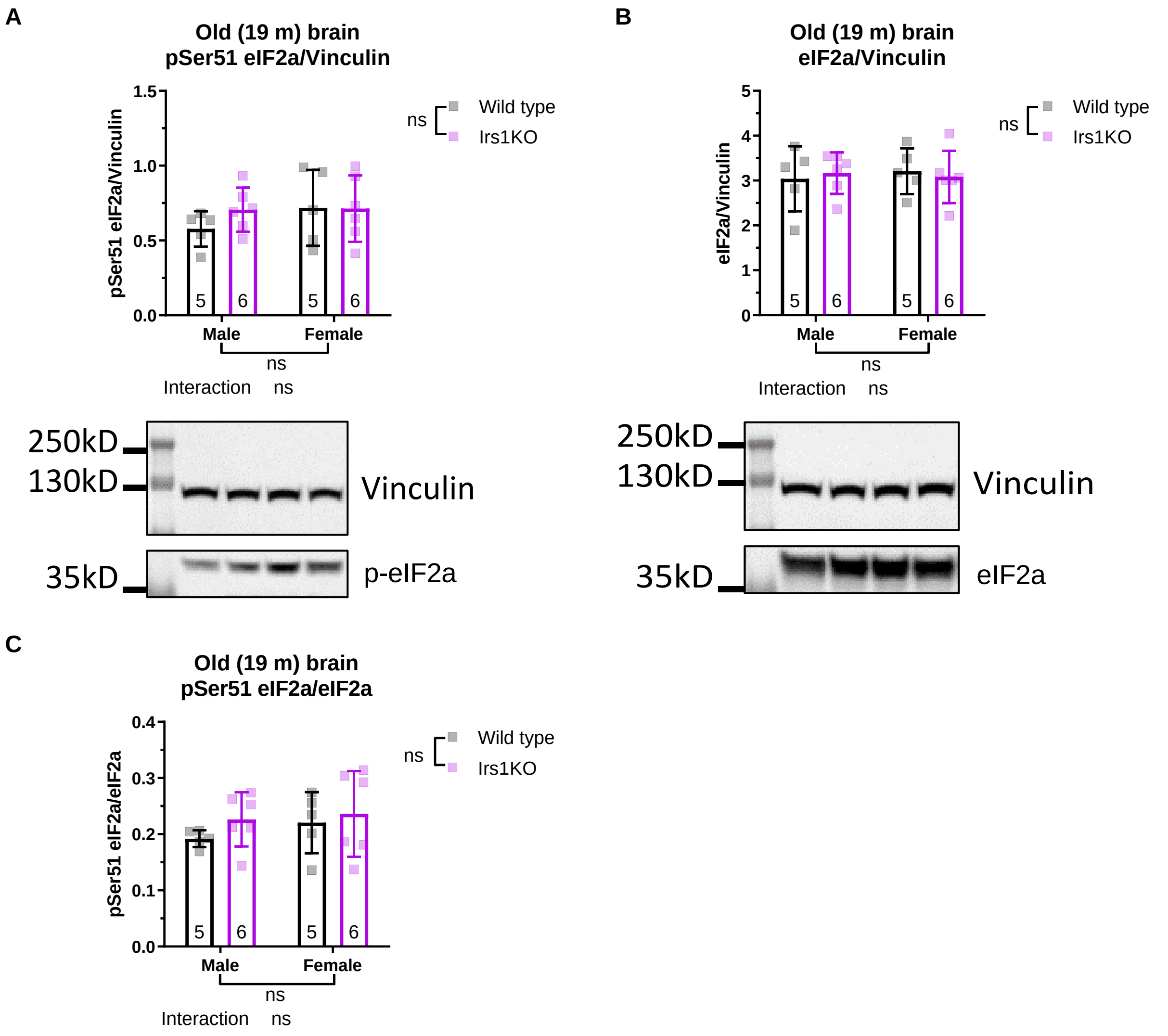

Supplementary Figure 15: ISR activation in Irs1KO brains is independent of eIF2a phosphorylation

Brain samples of old (19 months) Irs1KO mice showed no significant difference in phosphorylation of eIF2a at Serine 51 **(A)** or total eIF2a **(B)** as assessed by immunoblot. Moreover, normalising pSer51 eIF2a by total eIF2a did not reveal a significant difference in Irs1KO mice. Detailed statistical values found in Table S1. Raw immune blots in Table S3.

Supplementary Figure 16

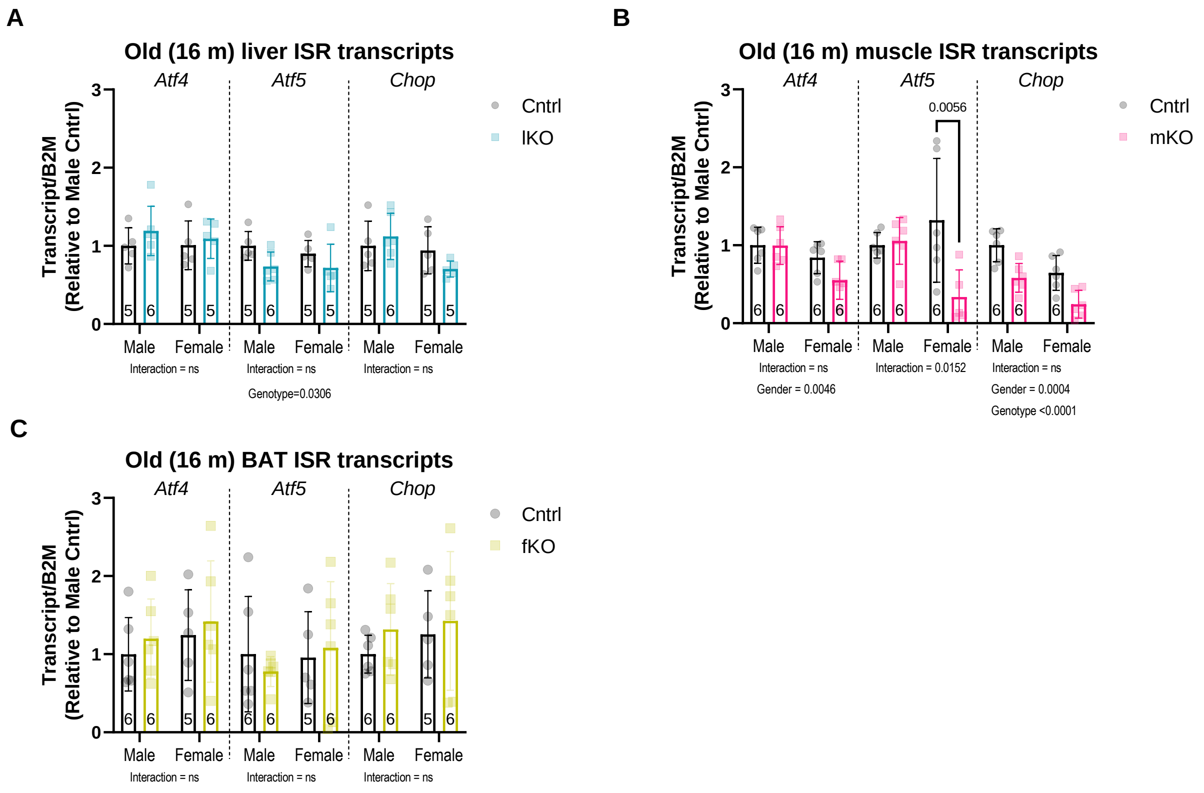

Supplementary Figure 16: IRS1 deletion in peripheral tissues is insufficient to induce local ISR signature in old mice

Quantitative real-time PCR performed on metabolic organs of *Irs1* tissue-specific deletion in old (16 months) mice targeting various integrated stress response (ISR) markers. **(A)** No significant difference in ISR transcripts was detected in the liver of lKO mice. **(B)** No significant increase in ISR transcript levels in hind limb muscle tissue of mKO mice, however we did detect a sex-specific downregulation of Atf5 transcripts in female mKO mice and genotype specific downregulation of Chop levels in mKO mice. **(C)** No difference in ISR transcripts was found in supraclavicular brown adipose tissue (BAT) of fKO mice. Detailed statistical values found in Table S1.

Supplementary Figure 17

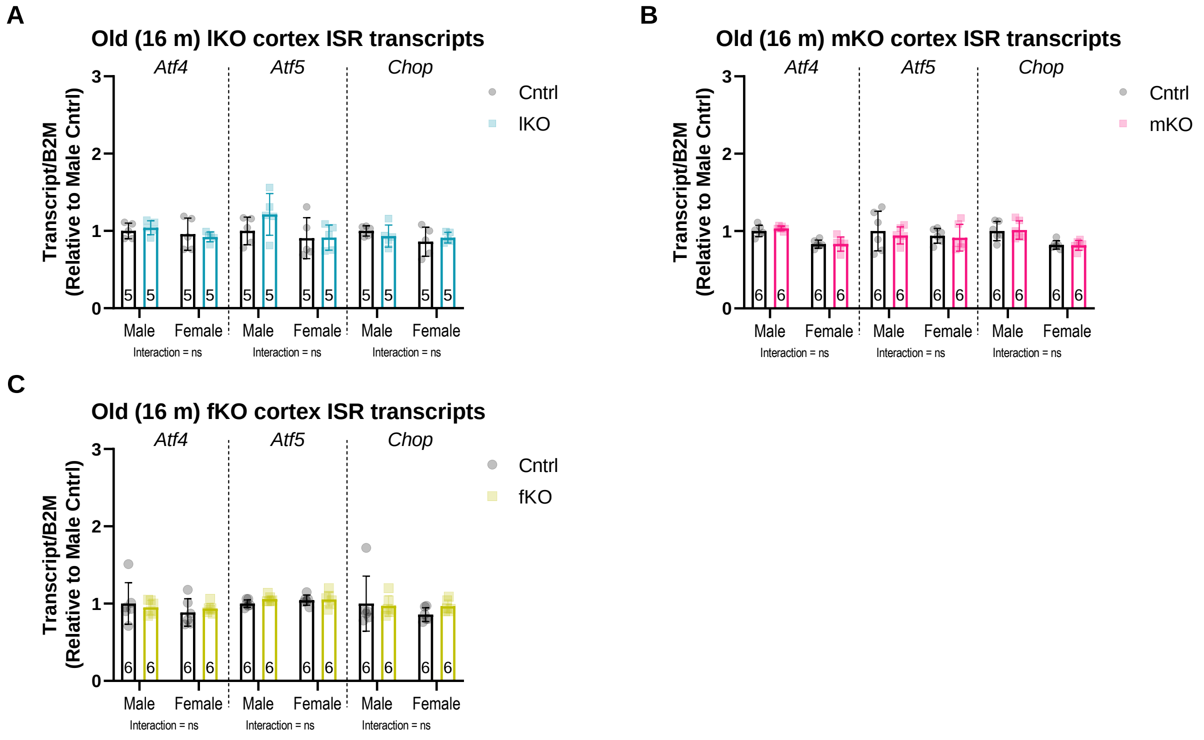

Supplementary Figure 17: IRS1 deletion in peripheral tissues does not induce ISR signature in brains of old mice

Quantitative real-time PCR performed on cortex samples of *Irs1* tissue-specific deletion in old (16 months) mice targeting various integrated stress response (ISR) markers. No significant difference in ISR transcripts was detected in **(A)** lKO, **(B)** mKO, or **(C)** fKO mice. Detailed statistical values found in Table S1.

Supplementary Figure 18

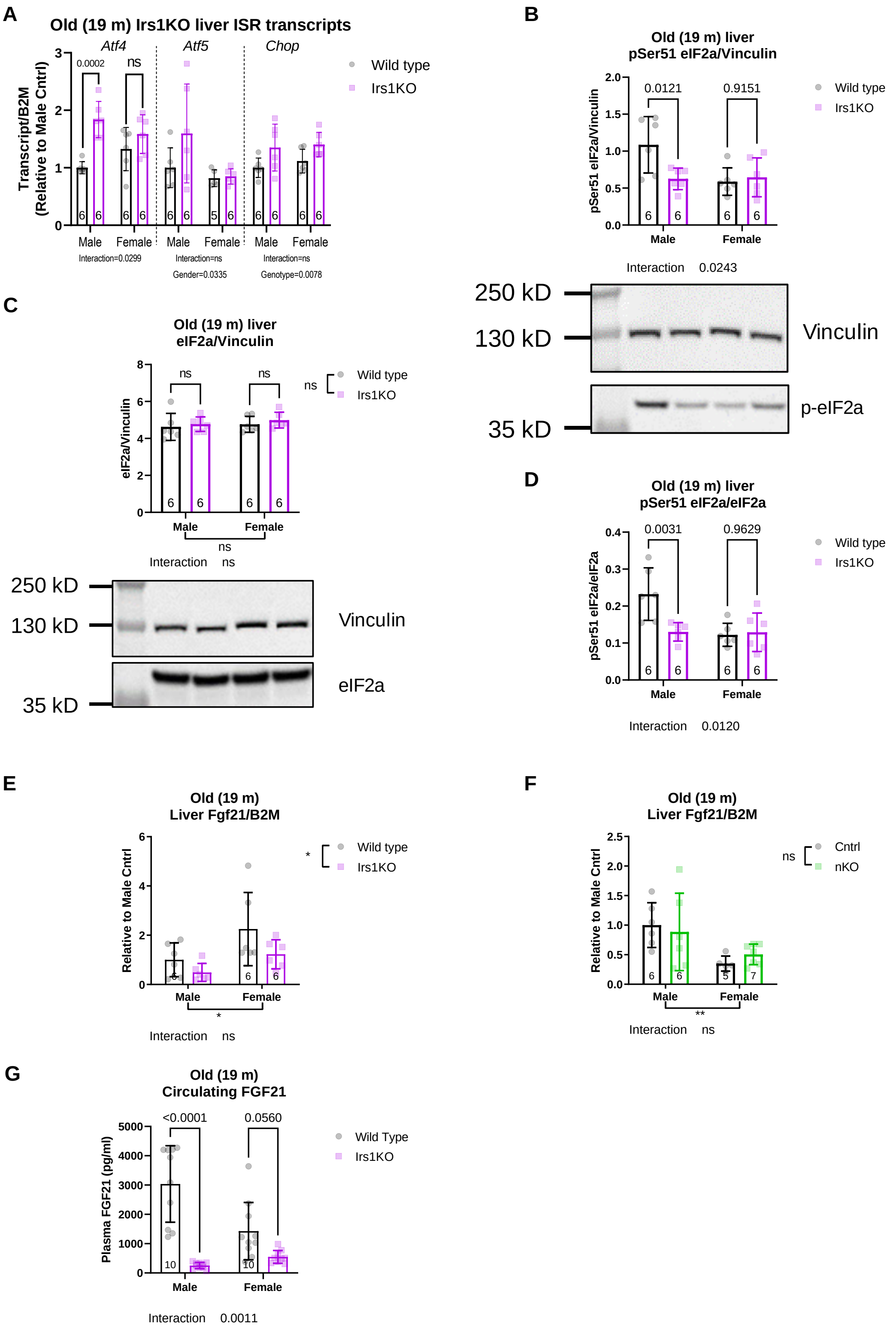

Supplementary Figure 18: Sex-specific brain ISR signal does not lead to a systemic FGF21 signal (below)

(A) Liver integrated stress response (ISR) transcripts of old (19 months) Irs1KO mice and their wild type littermates reveal sex-specific upregulation in male Irs1KO Atf4 levels, as well as a significant difference due to genotype in Chop levels. **(B)** Immunoblot analysis of hepatic levels of phosphorylated eIF2a revealed a sex-specific reduction in male Irs1KO mice. **(C)** Levels of total eIF2a did not show any significant difference in Irs1KO mice. **(D)** Normalising pSer51 eIF2a levels to total eIF2a revealed that pSer51 eIF2a levels were significantly reduced in a sex-specific manner in male Irs1KO mice. **(E)** Fgf21 transcript levels performed on liver tissue of old Irs1KO mice and their wild type littermates found a significant reduction of Fgf21 levels (n=6 biologically independent animals for all groups). **(F)** ELISA for FGF21 levels on plasma samples of old Irs1KO mice revealed significant reduction of plasma FGF21 levels, more so in male Irs1KO mice (male wild type and Irs1KO n=10, female wild type n=10 and Irs1KO n=9). **(G)** Fgf21 transcript levels in liver tissue of old nKO mice was not significantly different from their control littermates. Detailed statistical values found in Table S1. Raw immune blots in Table S3.

Supplementary Figure 19

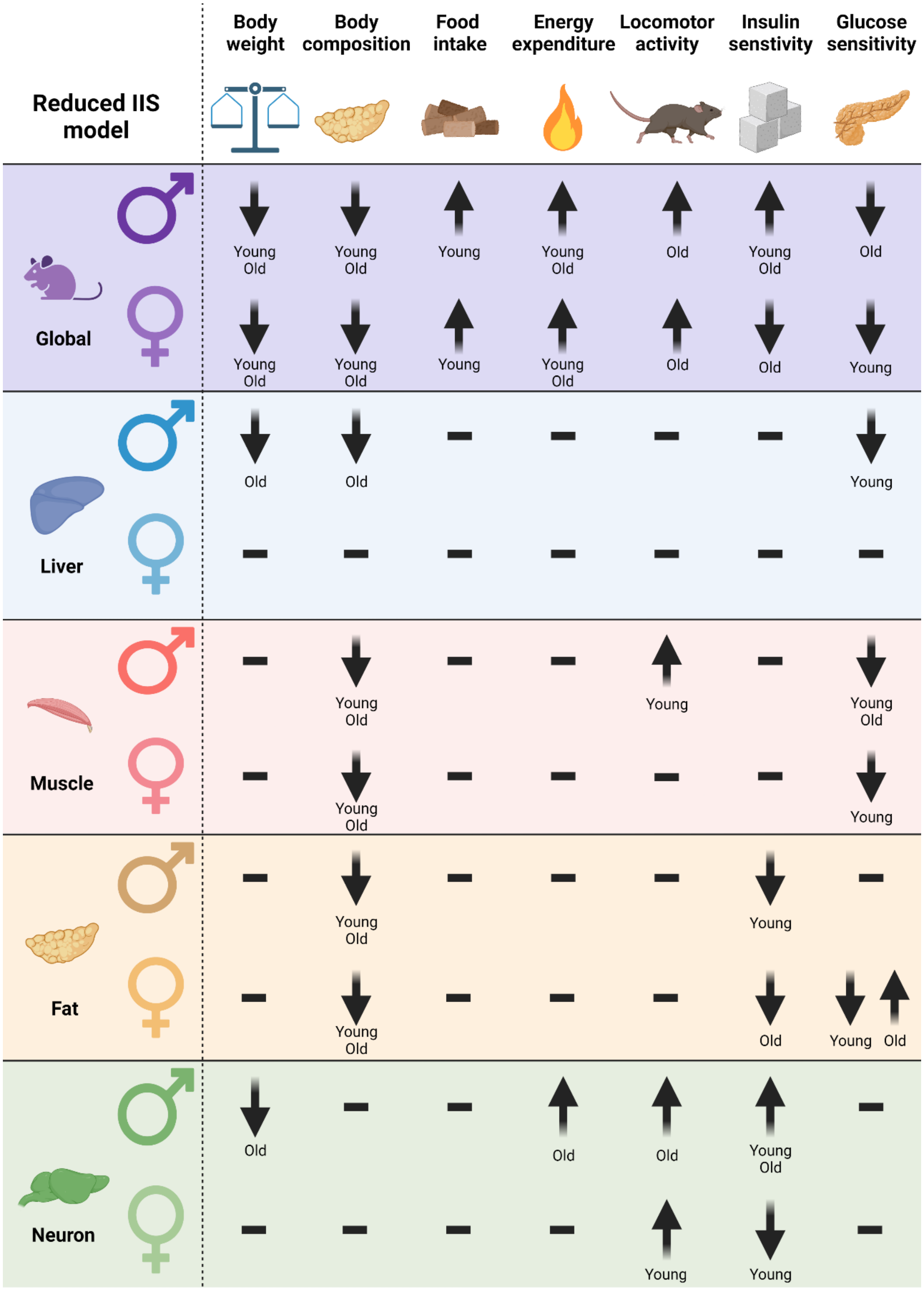

Supplementary Figure 19: Physiological outcomes present in a sex-specific and age-dependent manner in IRS1 deletion mutants

Graphical summary of physiological findings from the *Irs1* deletion models used in the study.

Supplementary Figure 20

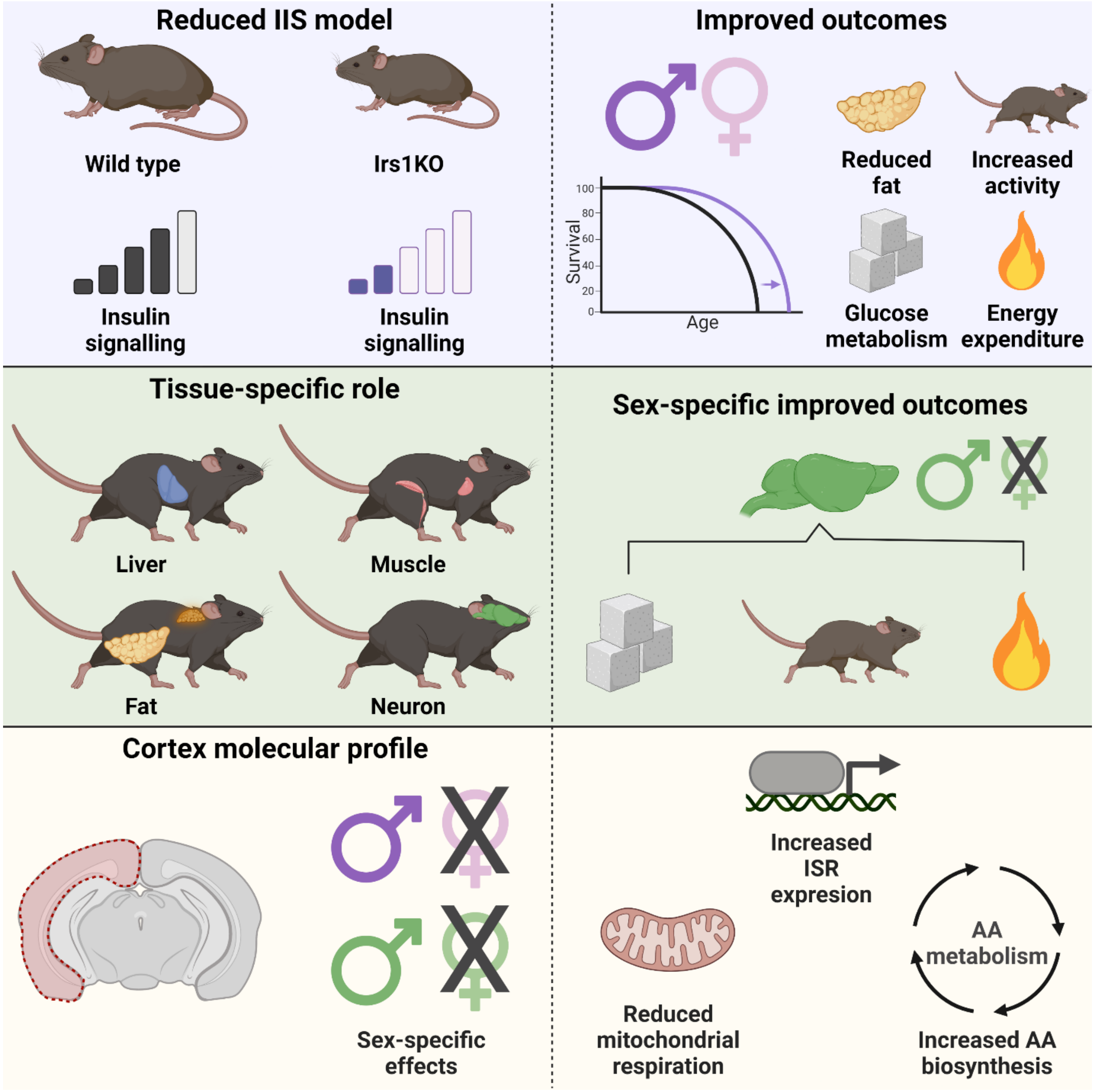

Supplementary Figure 20: Reduced insulin signalling in neurons induces sex-specific health benefits

Graphical abstract of the major findings in the study highlighting the replication of Irs1KO results, as well as finding sex-specific benefits in the neuronal IRS1 deletion mice. We identified a male-specific brain signature of ageing in response to IRS1 deletion implicating mitochondrial dysfunction that seems to be associated with improved health outcomes in old age.

Table S1: Detailed statistics for all main figures and supplementary material

Table S2: Raw data for semi-targeted metabolomics on Irs1KO and nKO brain tissue

Table S3: Supplementary information regarding primers, antibodies, and qPCR TaqMan probes used in the study

Table S4: Complete lifespan data for all mutant mice and their corresponding controls
